# Cytotoxicity of nimbolide towards multidrug-resistant tumor cells and hypersensitivity via cellular metabolic modulation

**DOI:** 10.18632/oncotarget.26299

**Published:** 2018-11-06

**Authors:** Nuha Mahmoud, Mohamed E.M. Saeed, Yoshikazu Sugimoto, Sabine M. Klauck, Henry J. Greten, Thomas Efferth

**Affiliations:** ^1^ Department of Pharmaceutical Biology, Johannes Gutenberg University, Mainz, Germany; ^2^ Division of Chemotherapy, Faculty of Pharmacy, Keio University, Tokyo, Japan; ^3^ Division of Cancer Genome Research, German Cancer Research Center, German Cancer Consortium, National Center for Tumor Diseases, Heidelberg, Germany; ^4^ Abel Salazar Institute of Biomedical Sciences, University of Porto, Porto, Portugal; ^5^ Heidelberg School of Chinese Medicine, Heidelberg, Germany

**Keywords:** HIF1α, limonoids, MDR, NF-κB, reactive oxygen species

## Abstract

Nimbolide is considered a promising natural product in cancer prevention and treatment. However, it is not known yet, whether the different mechanisms of multidrug resistance (MDR) influence its anticancer activity. In this study, well-known MDR mechanisms (*ABCB1*, *ABCG2*, *ABCB*5, *TP53*, *EGFR*) were evaluated against nimbolide. The P-glycoprotein (*ABCB1*/*MDR1*)-overexpressing CEM/ADR5000 cell line displayed remarkable hypersensitivity to nimbolide, which was mediated through upregulation of the tumor suppressor, PTEN, and its downstream components resulted in significant downregulation in *ABCB1*/*MDR1* mRNA and P-glycoprotein. In addition, nimbolide targeted essential cellular metabolic-regulating elements including HIF1α, FoxO1, MYC and reactive oxygen species. The expression of breast cancer resistance protein (BCRP) as well as epidermal growth factor receptor (EGFR) and mutant tumor suppressor *TP53* did not correlate to nimbolide’s activity. Furthermore, this paper looked for other molecular determinants that might determine tumor cellular response towards nimbolide. COMPARE and hierarchical cluster analyses of transcriptome-wide microarray-based mRNA expressions of the NCI 60 cell line panel were performed, and a set of 40 genes from different functional groups was identified. The data suggested NF-κB as master regulator of nimbolide’s activity. Interestingly, *HIF1α* was determined by COMPARE analysis to mediate sensitivity to nimbolide, which would be of great benefit in targeted therapy.

## INTRODUCTION

Despite the considerable efforts in the past decades to improve cancer treatment, the development of resistance towards anticancer therapy remains one of the most challenging situations. Cancer cells become resistant to various chemotherapeutic and xenobiotic drugs showing a trait known as multidrug resistance (MDR) [1]. Resistance even occurs in the most recent personalized anticancer therapies, which target specific molecular determinants in tumor cells [2]. The multiple factors behind cancer drug resistance clearly reflect its complexity. Host and tumor genetic alterations, signaling pathway alterations, epigenetic changes and tumor environment all seem to contribute to the fatal outcome of many tumor diseases [3–5]. Many relevant mechanisms for cytotoxic drug resistance have been described and discussed [1, 6, 7], among which the overexpression of the ATP-binding cassette (ABC) transporter proteins belongs to the most prominent MDR mechanisms. These membrane proteins represent one of the largest gene families in human beings and are responsible for the reduction of intracellular accumulation of many classical anticancer drugs below the effective level leading to drug resistance and treatment failure [8, 9]. P-glycoprotein (*ABCB1/MDR1*) is one of the most studied ABC transporters that are overexpressed in many tumor types [10]. P-glycoprotein consumes energy derived from ATP hydrolysis to expel an extremely broad range of structurally and functionally unrelated cytotoxic agents out of cancer cells such as taxanes (*e.g.* paclitaxel and docetaxel), *Vinca* alkaloids (*e.g.* vinblastine, vincristine, vindesine, vinorelbine), epipodophyllotoxins (*e.g.* teniposide, etoposide) and anthracyclines (*e.g.* doxorubicin, daunorubicin, epirubicin, idarubicin) [8]. Numerous inhibitors have been identified for P-glycoprotein’s efflux function [11–13]. Another well-known MDR-conferring ABC transporter is the breast cancer resistance protein (*ABCG2/*BCRP). It is associated with the MDR phenotype by active efflux to many approved conventional anticancer drugs and targeted small therapeutic molecules. For instance, doxorubicin, daunorubicin, mitoxantrone, and methotrexate are substrates for BCRP [8]. BCRP mediates MDR in breast, small cell lung, ovarian, colon, gastric or intestinal cancers. Besides, a strong correlation between high *ABCG2* expression and poor prognosis of leukemia patients has been described [14]. *ABCB5* is another ATP-binding MDR transporter that recently gathered attention. It mediates resistance to 7-Cl camptothecin and doxorubicin in human malignant melanoma [15]. Approaches of *ABCB5* blockade may provide therapeutic benefits, which are still under development.

It is apparent that more than one MDR mechanism can be present in cancer cells. The oncogenic gain of function of the tumor suppressor gene *TP53* due to the mutations is of great significance in cancer recurrence and resistance [16]. The accumulation of mutant *TP53* has been observed in many human tumors, and its contribution in the evolvement of cancer stem cells is noteworthy. The latter has been considered as tumor reservoir with self-protection characteristics that mediates MDR [17]. The role of mutant *TP53* for drug resistance may coincidence with its ability to mediate sustainable activation of the epidermal growth factor receptor (EGFR*)* pathway [18]. The expression of the *EGFR* gene occurs in a variety of tumors, including prostate, breast, gastric, colorectal, and ovarian carcinoma and affects treatment success [19]. Activation of *EGFR* signal transduction pathway leads to multiple biological processes such as gene expression and cellular proliferation, that eventually support tumor progression and promote oncogenesis [20]. More recently, TP53 has been recognized as treatment target to identify compounds that specifically target mutated *TP53* [16]. Another resistance mediator is the transcription factor nuclear factor kappa-light-chain-enhancer of activated B cells (NF-κB), which is a key regulator of immune and inflammatory responses. NF-κB regulates the expression of genes involved in the control of cellular proliferation and apoptosis [21]. The constitutive activation of NF-κB in some tumors enhanced the expression of anti-apoptotic and MDR genes, adding a new dimension to the MDR profile [22]. It is important to point out that tumor cells reprogram and modulate their signaling pathways to achieve metabolic adaptation, in order to rapidly proliferate and survive. Targeting cellular metabolism has been considered as novel strategy for cancer treatment [23].

New agents that are less susceptible to known resistance mechanisms or that even contribute to reverse drug resistance phenotypes are urgently needed. In this context, plant-derived compounds served as rich source for the development of novel therapeutic anticancer agents. Such evidently successful compounds are *Vinca* alkaloids from *Catharanthus roseus* G. Don. (Apocynaceae), the terpene paclitaxel from *Taxus brevifolia* Nutt. (Taxaceae), the lignan podophyllotoxin isolated from *Podophyllum peltatum* L. (Berberidaceae) and the DNA topoisomerase I inhibitor camptothecin from *Campototheca acuminata* Decne. (Nyssaceae). A promising medicinal plant in this area is *Azadirachta indica* (family: Meliceae), commonly known as Neem Tree. This tree is native to India and the Indian subcontinent with a wide distribution in tropical areas [24]. Nimbolide is one of the limonoids that has been isolated from Neem seeds and leaves. It has an interesting chemopreventive and therapeutic profile against tumor cells [25]. Remarkable cytotoxic effects were observed in cell lines derived from leukemia, colon cancer, prostate cancer, glioblastoma multiforme, breast cancer and others [26]. Nimbolide was found to induce anti-proliferation effect mediated by downregulation of cyclin-dependent kinases (CDKs) and/or cyclin molecules causing cell cycle arrest [27]. Induction of apoptosis through both intrinsic and extrinsic pathways has been reported [28]. Nimbolide also targets diverse signaling cascades such as MAPK (ERK1/2), PI3K/Akt, Wnt/β-catenin and JAK2/STAT3, resulting in cell growth abrogation and anticancer effect [29]. Moreover, literature evidence indicats that nimbolide reduces angiogenesis and migration, in addition to suppression of tumorigenesis [25, 30, 31].

To best of our knowledge, the activity of nimbolide towards MDR phenotypes has been not yet investigated. This study was designed to explore the cellular responsiveness of nimbolide in sensitive and drug-resistant cell lines that especially overexpress classical MDR genes, namely ABC transporter proteins (*ABCB1, ABCB5, ABCG2*), TP53 or EGFR. Since P-glycoprotein-overexpressing cells showed remarkable sensitivity compared to their sensitive counterparts in our investigations, we further explored the underlying molecular mechanisms. The effect of nimbolide towards NF-κB was also evaluated. Lastly, the NCI cell line panel was used to identify putative molecular factors that may determine the response of tumor cells to nimbolide.

## RESULTS

### Cytotoxicity assays for nimbolide towards MDR-expressing cell lines

**Figure 1:**
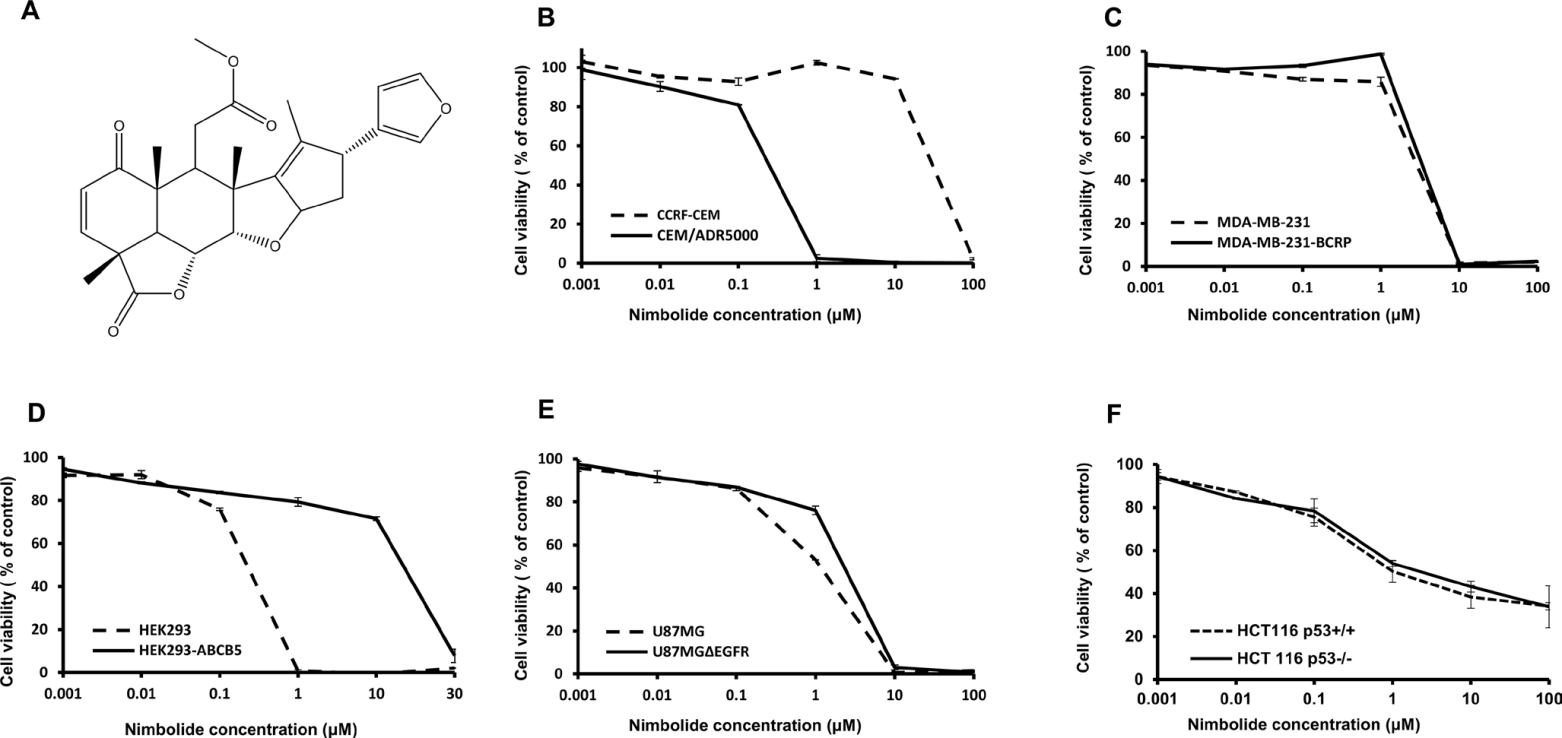
Nimbolide structure and cytotoxicity dose-response curves as determined by resazurin assay (**A**) Chemical structure. (**B**) Drug-sensitive CCRF-CEM and P-glycoprotein-overexpressing CEM/ADR5000 cells. (**C**) Sensitive MDA-MB-231 pcDNA and BCRP-transfected MDA-MB-231 cells. (**D**) Non-transfected HEK293 cells and ABCB5-transfectant HEK293 subline. (**E**) Wild-type U87.MG cells and their mutated EGFR-transfected subline, U87.MGΔEGFR. (**F**) HCT116 p53^+/+^ and their knockout p53^−/−^ subline. The curves show mean values ± SD of three independent experiments with each 6 parallel measurements.

Different concentrations of nimbolide (from 0.001 μM to 100 μM) were used to perform resazurine assays. Interesting results came from testing the sensitivity of multidrug-resistant *ABCB1/MDR1*-expressing CEM/ADR5000 and parental CCRF-CEM leukemia cells. Nimbolide showed hypersensitivity towards CEM/ADR5000 cells with an IC_50_ value of 0.3 (± <0.01) μM compared to 17.4 (± 0.6) μM in CCRF-CEM cells, displaying a phenomenon known as collateral sensitivity [32]. The nimbolide activity was also tested in breast cancer cells transduced with cDNA for *ABCG2/*BCRP and compared with sensitive cells transduced with a control vector. The IC_50_ values for sensitive and resistant cell lines were 4.7 (± 0.05) μM and 3.7 (± 0.2) μM, respectively, indicating that cellular responsiveness to nimbolide may not be relevant to *ABCG2/*BCRP expression. In HEK293 cells, nimbolide was unexpectedly recognized as a substrate for *ABCB5*-transfected cells, with an IC_50_ value of 14.5 (± 0.3) μM compared to 0.25 (± 0.02) μM for the sensitive non-transfected ones. Furthermore, the cytotoxicity curves of nimbolide against glioblastoma and colon sensitive and resistant cell lines were comparable (1.12 (± <0.01) μM) for U87.MG and 3.4 (± 0.1) μM for U87.MGΔEGFR, 0.9 (± 0.05) μM for HCT116 p53^+/+^ and 1.8 (± 0.1) μM for HCT116 p53^−/−^). Hence, EGFR and TP53 may not be related to nimbolide activity. All concentration-dependent curves are depicted in Figure 1.

### Nimbolide-induced collateral sensitivity in P-glycoprotein-overexpressing cells

### Functional pathway analysis

**Figure 2:**
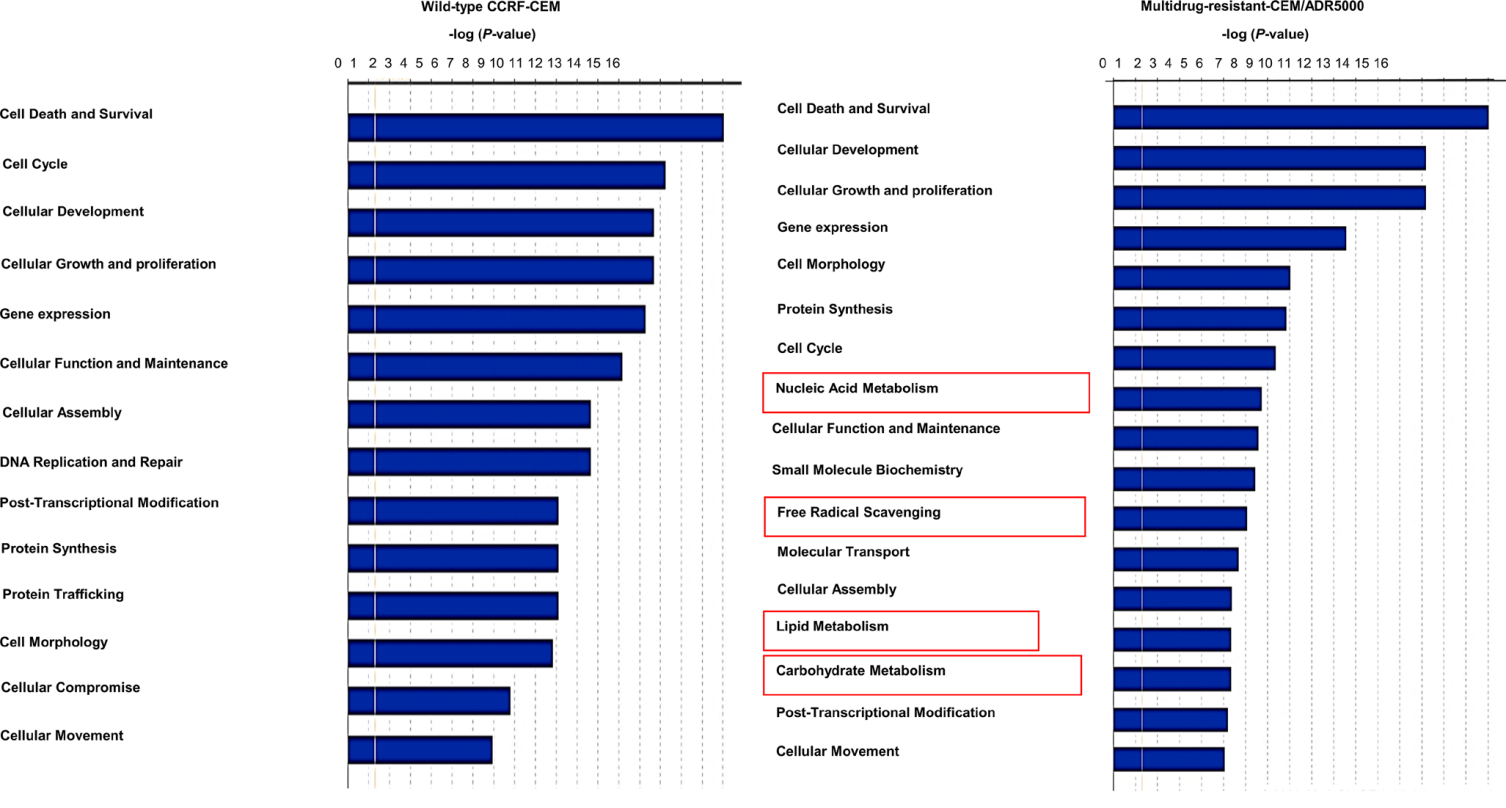
Ingenuity pathway analysis Top cellular and molecular functions affected by nimbolide in CCRF-CEM and P-glycoprotein-overexpressing CEM/ADR5000 cells.

**Figure 3:**
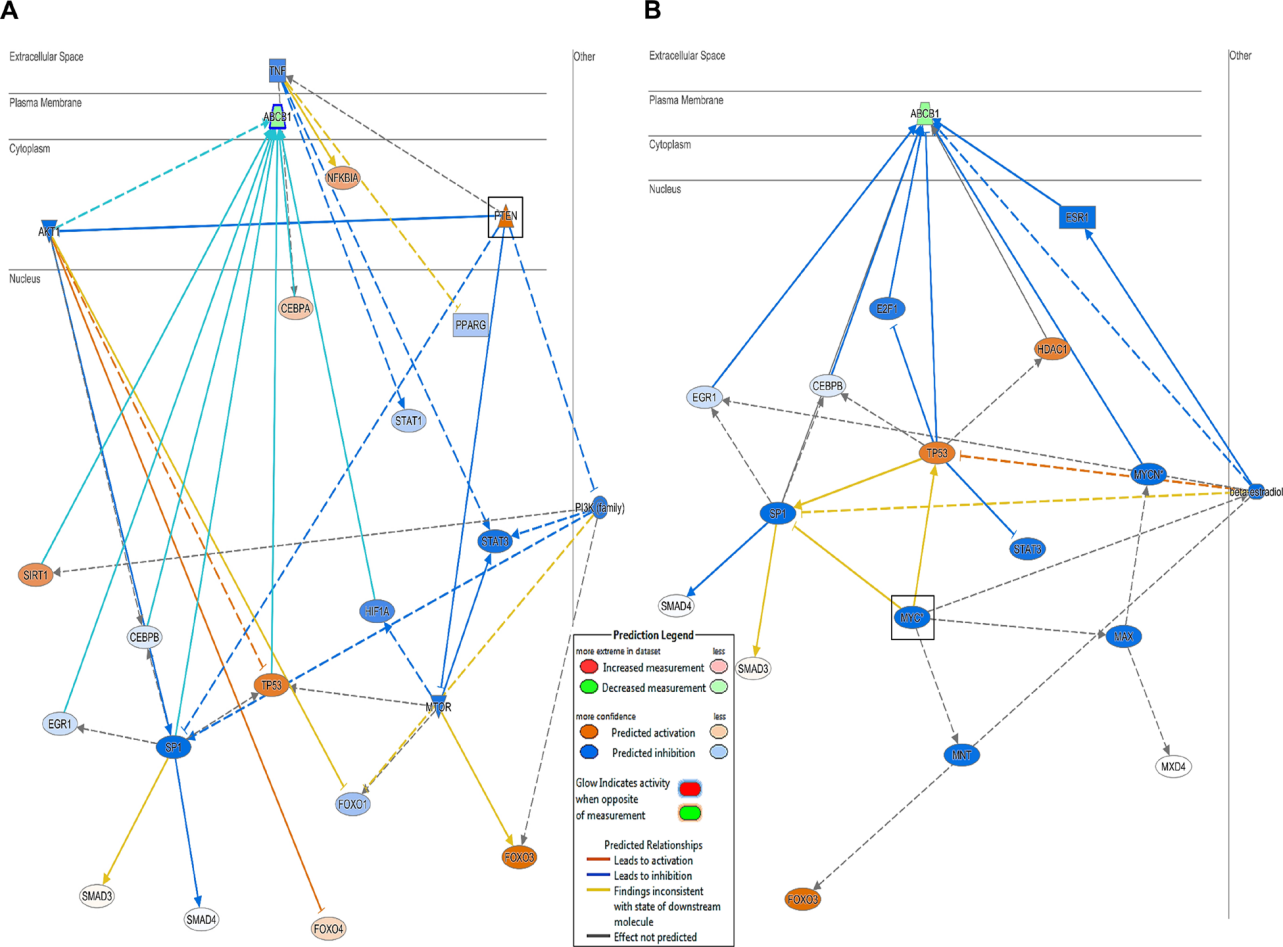
PTEN and MYC downstream effect and molecular network Upstream analysis for P-glycoprotein-overexpressing cells identified (**A**) PTEN as the most significantly activated regulator (*P =* 7.98 × 10^−13^) and (**B**) MYC as the most significantly inhibited regulator (*P =* 3.89 × 10^−26^).

Microarray-based gene expression data was analyzed using bioinformatics tools. Chipster analyses identified 492 and 731 significantly deregulated genes in CCRF-CEM and CEM/ADR5000 cells, respectively (see Supplementary Tables 1 and 2). In both cell lines, the Ingenuity Pathway Analysis (IPA) program predicted cell death and survival, cellular development and cellular growth and proliferation as the most affected molecular and cellular functions (Figure 2). Nucleic acid metabolism, lipid metabolism, carbohydrate metabolism and free radical scavenging were only modulated in resistant cells and linked to their hypersensitivity response towards nimbolide. Remarkably, upstream analysis for P-glycoprotein-overexpressing cells identified PTEN and MYC as the most significantly targeted upstream regulators (Figure 3). Genes in CEM/ADR5000 cells that are consistent with activation by PTEN (*P =* 7.98 × 10^−13^) or inhibition by MYC (*P =* 3.89 × 10^−26^) are shown in Tables 1 and 2.

**Table 1:** Genes in P-glycoprotein-overexpressing CEM/ADR5000 cells those are consistent with PTEN activation after nimbolide treatment

Gene symbol	Exper. Fold change	Gene symbol	Exper. Fold change
*MXD4*	2.325	*CXCR4*	−1.095
*KLHL24*	1.800	*FAS*	−1.115
*NDRG1*	1.720	*CFD*	−1.185
*MEF2D*	1.290	*CCND1*	−1.185
*AKR1C3*	1.255	*MAPKAPK3*	−1.200
*SAT1*	1.215	*PA2G4*	−1.270
*HSPA1A/HSPA1B*	1.215	*NOP2*	−1.320
*TOM1*	1.175	*SCD*	−1.345
*IFRD1*	1.170	*VEGFB*	−1.515
*BTG1*	1.090	*CDK4*	−1.590
*KLF6*	1.005	*ATP5MC1*	−1.680
*ACACA*	−1.010	*MRPL12*	−2.145
*H2AFY*	−1.015	*FASN*	−2.355
*SREBF1*	−1.025	*MYC*	−4.260
*SORD*	−1.065		

IPA program highlighted 29 genes from the dataset that have measurement directions in consistence with PTEN activation (*p*-*value* 7.98 × 10^−13^).

**Table 2:** Genes in P-glycoprotein-overexpressing CEM/ADR5000 cells those are consistent with MYC inhibition after nimbolide treatment

Gene symbol	Exper. Fold change	Gene symbol	Exper. Fold change	Gene symbol	Exper. Fold change
*DDIT3*	2.285	*HNRNPU*	−1.225	*HK2*	−1.545
*IRF7*	1.815	*PPAT*	−1.235	*PTPRC*	−1.580
*NDRG1*	1.720	*KAT2A*	−1.235	*CDK4*	−1.590
*CCNG2*	1.560	*TFRC*	−1.240	*RPL6*	−1.605
*SCPEP1*	1.300	*NOP58*	−1.260	*PTMA*	−1.625
*FTH1*	1.290	*TYMS*	−1.265	*CCND2*	−1.625
*SAT1*	1.215	*ID1*	−1.265	*SUMO3*	−1.690
*ALB*	1.085	*PA2G4*	−1.270	*MCM7*	−1.690
*MCM6*	−1.010	*RRM2*	−1.290	*DCTPP1*	−1.700
*ACACA*	−1.010	*MIF*	−1.295	*SRM*	−1.720
*HSPA9*	−1.020	*AK2*	−1.310	*PRMT1*	−1.760
*RRP1B*	−1.030	*RPS7*	−1.325	*DDX21*	−1.765
*CAD*	−1.035	*DDX39B*	−1.330	*PAICS*	−1.780
*PPP2CA*	−1.065	*ALDH18A1*	−1.345	*RRS1*	−1.810
*POU4F1*	−1.080	*TPI1*	−1.365	*ABCE1*	−1.820
*NME2*	−1.085	*BCAT1*	−1.370	*GART*	−1.845
*CDK6*	−1.090	*DNPH1*	−1.380	*ABCB10*	−1.845
*CD44*	−1.110	*CDCA7*	−1.395	*NME1*	−1.850
*EFTUD2*	−1.115	*C1QBP*	−1.395	*IRX3*	−1.850
*MIR17HG*	−1.150	*PRDX4*	−1.400	*NOLC1*	−1.875
*PPIA*	−1.180	*NOP56*	−1.410	*PHB*	−2.035
*ODC1*	−1.180	*PGAM1*	−1.415	*MRPL12*	−2.145
*ENO1*	−1.190	*SNRPD1*	−1.435	*RANBP1*	−2.225
*EIF4A1*	−1.190	*LDHA*	−1.475	*FASN*	−2.355
*PRDX2*	−1.200	*BZW2*	−1.500	*PFAS*	−2.375
*ATAD3A*	−1.215	*HNRNPAB*	−1.535		

IPA program highlighted 77 genes from the dataset that have measurement directions in consistence with MYC inhibition (*p-value* 3.89 × 10^−26^).

### Validation of relative genes expression by quantitative reverse transcription PCR

**Figure 4:**
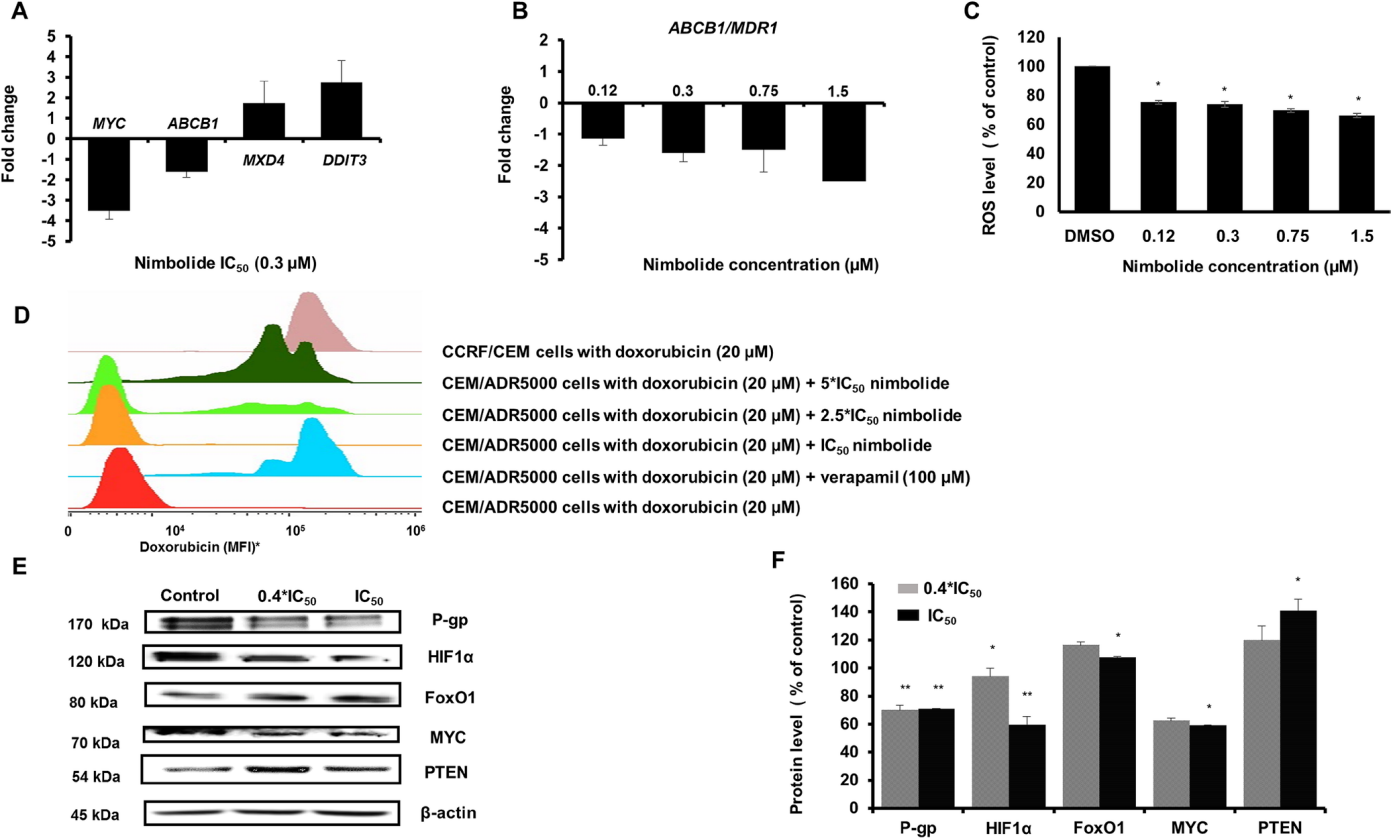
(**A**) The effect of nimbolide IC_50_ on *MYC, ABCB1*, *MXD4,* and *DDIT3* expression levels. Strong correlation between microarray data and qPCR was confirmed with *R*-value = 0.98 (Table 3). (**B**) The effect of different concentrations of nimbolide (0.4^*^IC_50_, IC_50_, 2.5^*^IC_50_ and 5^*^IC_50_) on the expression of *ABCB1/MDR1.* (**C**) Significant reduction of reactive oxygen species in CEM/ADR5000 cells after treatment with different concentrations of nimbolide. The results show mean values ± SD of three independent experiments. (^*^p < 0.05, compared to DMSO control cells). (**D**) The uptake of 20 μM doxorubicin was measured after 24 h in the presence of different concentrations of nimbolide and compared to doxorubicin uptake in CCRF-CEM. Verapamil 100 μM, a known P-glycoprotein inhibitor was used as a positive control. (^*^MFI, mean fluorescence intensity). (**E** and **F**) The effect of nimbolide on the protein expression levels for P-glycoprotein, HIF1α, FoxO1, MYC and PTEN in CEM/ADR5000 cells. The bands were normalized to β-actin and the mean values ± SD are shown in the results. (^*^p < 0.05, ^**^p < 0.01compared to DMSO control cells).

We selected four genes related to the obtained functional pathway analysis in CEM/ADR5000 cells to validate their relative expressions with qPCR. The expression of *MYC, MXD4, ABCB1* and *DDIT3* was normalized to *GAPDH*, and the fold-change values of microarray hybridization and qPCR were then subjected to Pearson correlation test. With an *R*-value equal to 0.98, we assured the high conformity of microarray and qPCR data (Table 3 and Figure 4A). We particularly investigated the effect of different concentrations of nimbolide on the expression of *ABCB1/MDR1.* The data demonstrated a dose-dependent down-regulation (Figure 4B).

**Table 3:** Validation of microarray relative genes expression by quantitative real-time reverse transcription PCR

Gene name	Microarray data (FC)^*^	qPCR data (FC)
*MYC*	−4.3	−3.5 ± 0.4
*ABCB1/MDR1*	−1.3	−1.6 ± 0.3
*MXD4*	2.3	1.1 ± 1.1
*DDIT3*	2.3	2.8 ± 1.3

The *R*-value of 0.98, indicated a strong correlation coefficient between fold-change (^*^FC) values obtained from microarray hybridization and qPCR.

### Uptake assay and ROS quantification

The effect of nimbolide on the efflux pump activity in P-glycoprotein-overexpressing cells was examined using flow cytometry. The results revealed increased cellular retention of doxorubicin after nimbolide treatment compared to untreated cells and similar to verapamil, which was used as control drug. It is well known that verapamil acts as a chemosensitizing agent and increases intracellular levels of cytotoxic P-glycoprotein substrates through its ability to stimulate ATPase activity, leading to ROS-mediated cell death. By contrast, nimbolide detoxified ROS cellular baseline suggesting another mechanism for collateral sensitivity (Figure 4C and 4D).

### Western blot

From previous findings, IPA particularly recognized nimbolide induced-alterations in the metabolic pathways in P-glycoprotein-overexpressing cells (see Figure 2). Besides, the upstream analysis identified PTEN, MYC, HIF1α and FoxO1 as the most affected metabolic regulators. This prompted us to further test the effect of nimbolide on their protein expression levels, and to examine, whether nimbolide indeed induced-collateral sensitivity by targeting the cellular metabolism. The protein expression levels of MYC and HIF1α were down-regulated and those of PTEN and FoxO1 were up-regulated (Figure 4E and 4F).

### COMPARE and hierarchical cluster analyses

COMPARE analysis was performed to recognize any novel molecular determinants that might contribute to the different sensitivity shown in Figure 1. Top genes with positive and negative correlations were identified and shown in Table 4. These genes belong to different functional groups such as those encoding ribosomal proteins and components of the mitochondrial respiratory chain, in addition to proteins with anti-apoptotic functions, DNA repair, replication, cell proliferation regulators and others. Interestingly, *HIF1α* was determined as a sensitivity factor of nimbolide.

**Table 4:** COMPARE coefficient obtained from the correlation of the mRNA expression of genes in NCI cell line panel with log_10_IC_50_ values of nimbolide

Coefficient	Exper. ID	Gene bank accession	Gene symbol	Name	Function
0.681	GC37319	W52024	*RPS15A*	Ribosomal protein S15a	Ribosomal protein
0.651	GC34926	X79563	*RPS21*	Ribosomal protein S21	Ribosomal protein
0.648	GC31906	AF070071	*MSH5*	MutS homolog 5 (*E. coli*)	Involved in meiotic recombination
0.646	GC31589	T89651	*RPL36A*	Ribosomal protein L36a	Ribosomal protein
0.637	GC29092	AA733050	*SNRPE*	Small nuclear ribonucleoprotein polypeptide E	Component of the pre-mRNA processing spliceosome
0.636	GC39380	U09953	RPL9	Ribosomal protein L9	Ribosomal protein
0.636	GC30848	X74262	*RBBP4*	Retinoblastoma binding protein 4	Involved in histone acetylation and chromatin assembly.
0.633	GC36012	AI095013	*HIST1H2AM*	Histone cluster 1. H2am	Core component of nucleosome
0.633	GC27139	D63482	*GIT2*	G protein-coupled receptor kinase interacting ArfGAP 2	GTPase-activating
0.619	GC28890	Y07969	*ANP32B*	Acidic (leucine-rich) nuclear phosphoprotein 32 family. member B	Cell cycle progression factor
0.619	GC30164	AF054187	*NACA*	Nascent polypeptide-associated complex α subunit	Involved in cell signaling
0.615	GC35423	X53777	*RPL17*	Ribosomal protein L17	Ribosomal protein
0.614	GC36655	U14966	*RPL5*	Ribosomal protein L5	Ribosomal protein
0.612	GC36652	L38941	*RPL34*	Ribosomal protein L34	Ribosomal protein
0.608	GC35734	AI557852	*RPS27*	Ribosomal protein S27	Ribosomal protein
0.606	GC27422	J02923	*LCP1*	Lymphocyte cytosolic protein 1 (L-plastin)	Actin-binding protein
0.602	GC32838	D55716	*MCM7*	Minichromosome maintenance complex component 7	Essential for genome replication
0.602	GC36916	AJ223349	*HIRIP3*	HIRA interacting protein 3	Role in chromatin function and histone metabolism
0.597	GC31486	X95525	*TAF5*	TATA box binding protein (TBP)-associated factor	Regulation of RNA polymerase transcription
0.595	GC39124	AA526497	*UQCRH*	Ubiquinol-cytochrome c reductase hinge protein	Part of the mitochondrial respiratory chain
−0.569	GC31852	AF037339	*CLPTM1*	Cleft lip and palate associated transmembrane protein 1	Role in T-cell development
−0.559	GC37799	M22299	*PLS3*	Plastin 3	Role in the regulation of bone development
−0.54	GC31915	N36926	*GNA11*	Guanine nucleotide binding protein (G protein). α 11 (Gq class)	Involved in transmembrane signaling
−0.534	GC32200	AL096879	*TMEM184B*	Transmembrane protein 184B	Activates the MAP kinase signaling pathway
−0.531	GC30688	AL009266	*RBFOX2*	RNA binding motif protein 9	Regulator of exon splicing in the nervous system
−0.525	GC32662	D00017	*ANXA2*	Annexin A2	Involved in transduction pathways
−0.517	GC29447	AJ133534	*RABAC1*	Rab acceptor 1 (prenylated)	Controls vesicle docking and fusion
−0.503	GC29436	D14696	*LAPTM4A*	Lysosomal protein transmembrane 4 α	Important for nucleosides transportation
−0.496	GC32112	U97018	*EML1*	Echinoderm microtubule associated protein like 1	Required for neuronal progenitor cells proliferation
−0.496	GC30594	AF038187	*WSB2*	WD repeat and SOCS box-containing 2	Component of ubiquitination processes
−0.49	GC36562	U48861	*CHRNB4*	Cholinergic receptor. nicotinic β4	Extracellular ligand-gated ion channel activity
−0.489	GC33491	L77886	*PTPRK*	Protein tyrosine phosphatase. receptor type. K	Negative regulator of EGFR signaling pathway
−0.486	GC38772	AF089816	*GIPC1*	GIPC PDZ domain containing family. member 1	Involved in G protein-linked signaling
−0.486	GC38406	AB011541	*MEGF8*	Multiple EGF-like-domains 8	Unknown function
−0.486	GC33008	U22431	*HIF1A*	Hypoxia inducible factor 1. α subunit (basic helix-loop-helix transcription factor)	Master transcriptional regulator of the adaptive response to hypoxia
−0.481	GC31510	AF062006	*LGR5*	Leucine-rich repeat-containing G protein-coupled receptor 5	Role in the formation of adult intestinal stem cells
−0.477	GC34135	M35011	*ITGB5*	Integrin β5	Receptor for adenovirus type C
−0.47	GC32475	M61916	*LAMB1*	Laminin β1	Mediates cells differentiation into tissues during embryonic development
−0.469	GC29175	AA487755	*FKBP9*	FK506 binding protein 9	Accelerates protein folding
−0.468	GC37524	AJ012582	*HCN2*	Hyperpolarization activated cyclic nucleotide-gated potassium channel 2	Contributes to spontaneous rhythmic activity in heart and brain

Information on gene functions was taken from the GeneCards database of the Weizman Institute of Science (https://www.genecards.org/)_._

**Figure 5:**
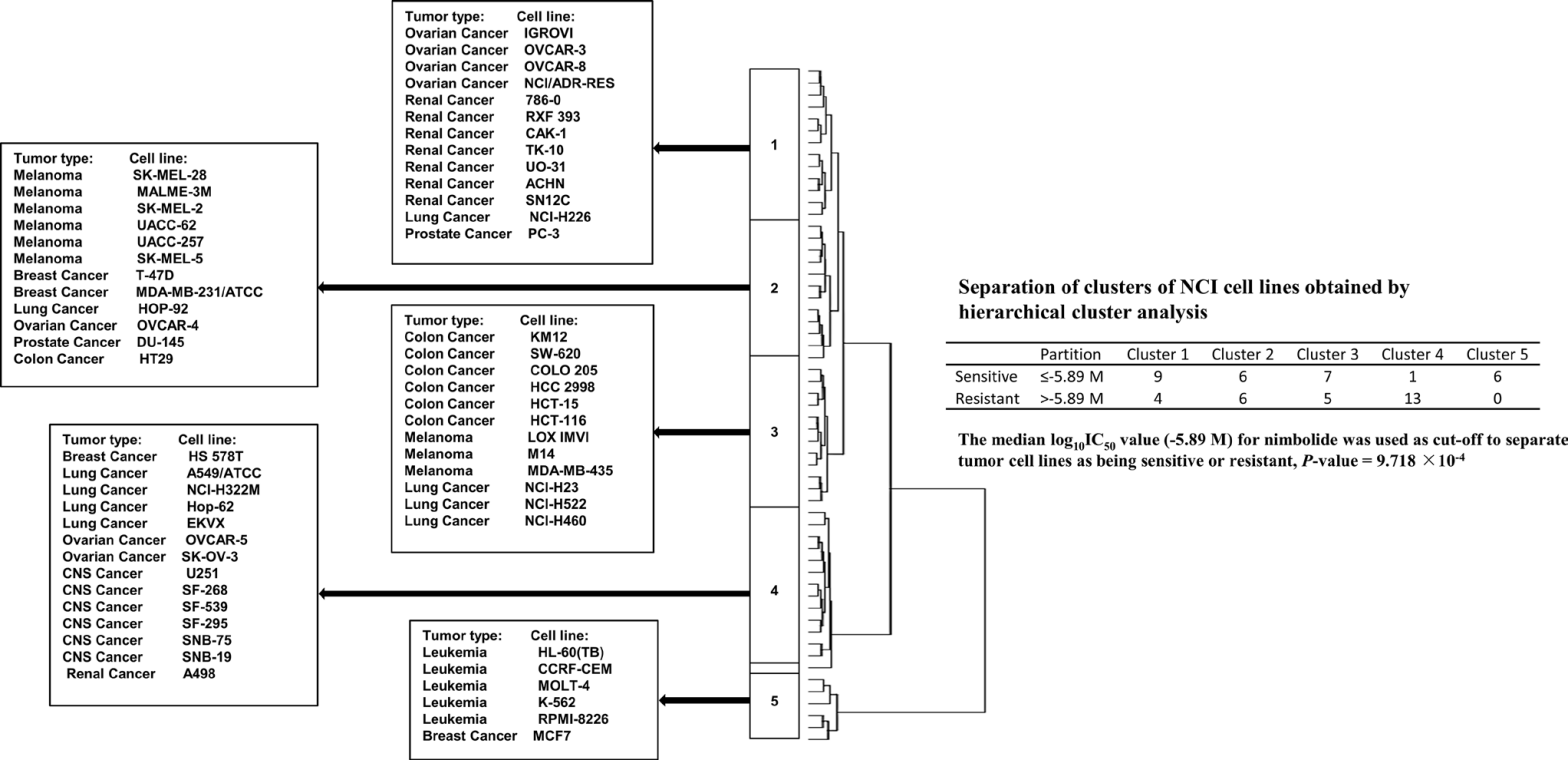
Hierarchical cluster analysis of microarray-based mRNA expression of genes obtained by COMPARE analysis The dendrogram shows the clustering of the cell line panel into five main branches and indicates the degrees of relatedness between cell lines.

To analyze whether the gene expression profiles of the 60 cell lines might predict sensitivity or resistance to nimbolide, hierarchical cluster analyses were applied to mRNA expression data and five main cluster branches appeared with a statistically significant relationship (*P* = 9.718 ×10^−4^, Figure 5).

### Transcription factor binding motif analysis and NF-kB reporter assay

**Figure 6:**
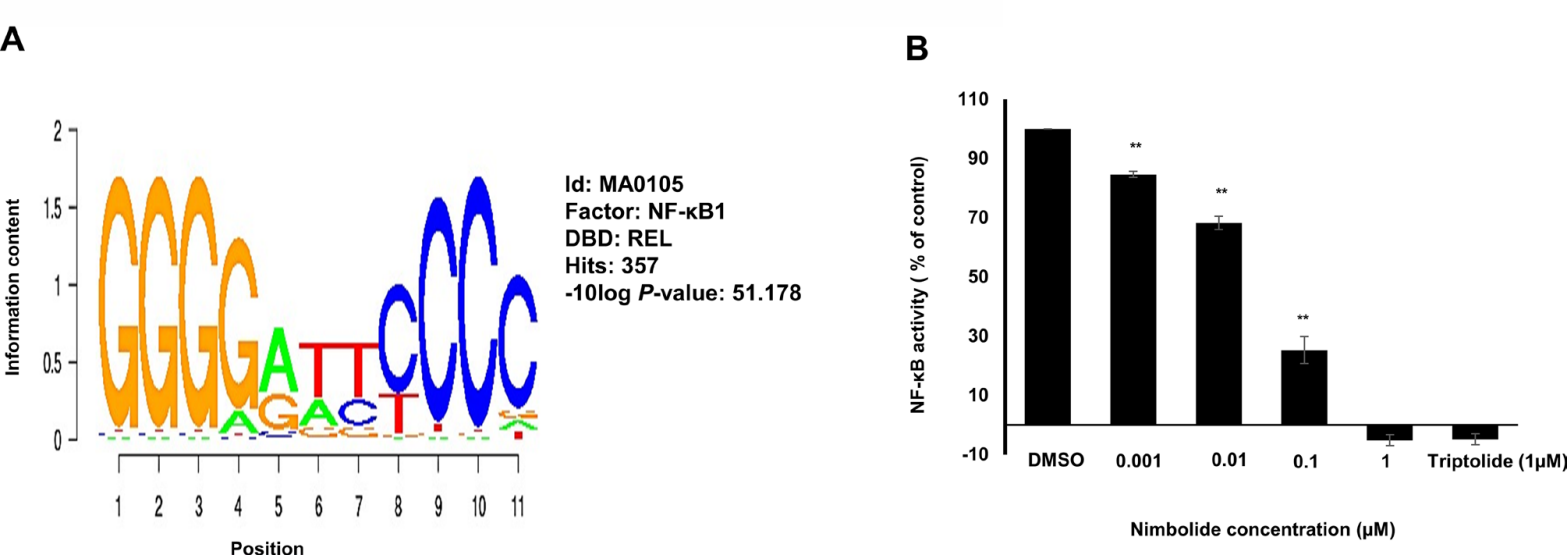
Nimbolide inhibits NF-κB activity (**A**) Motif screening of 25 kb upstream regions of 40 genes identified by COMPARE analysis revealed the significant presence of NF-κB-DNA binding motifs. (**B**) Various concentrations of nimbolide (0.001 μM. 0.01 μM. 0.1 μM and 1 μM) were used to treat SEAP-driven cells stimulated with TNF-α for 24 h and compared to the positive control triptolide (1 μM). The results show mean values ± SD of three independent experiments. (^**^p< 0.01, compared to DMSO control cells).

The genes that have been identified by COMPARE analysis (see Table 4) were suggested to be regulated by common transcription factors. Therefore, transcription factor binding motif analysis was performed to 25 kb upstream promoter sequence of the 40 genes, and the results revealed significant presence of NF-κB-DNA binding motifs with 357 hits (Figure 6A).

A secreted alkaline phosphatase (SEAP)-driven NF-κB reporter cell line was used to investigate the effect of nimbolide towards the transcription factor NF-κB. Nimbolide significantly inhibited NF-κB activity at a low concentration of 0.1 μM after 1 h. Triptolide was used as positive control drug for NF-κB inhibition (Figure 6B).

## DISCUSSION

### Cytotoxicity of nimbolide against MDR-expressing cells

This study is part of an integrated strategy for discovering novel agents from natural sources to circumvent MDR. One of the first mechanisms to be investigated is the role of P-glycoprotein-mediated MDR. The fact that P-glycoprotein-overexpression has been detected in more than 50% of human cancers and has been correlated to inherent and acquired MDR led to intensified efforts to inhibit P-glycoprotein’s function [10]. P-glycoprotein-targeting chemosensitizers were designed to inactivate this transporter. However, they have experienced difficulties to pass clinical trials [33]. This has raised interest searching for novel agents that selectively kill *ABCB1/MDR1-*expressing cells as alternative strategy to overcome MDR through tumor re-sensitization [34]. Such agents show greater sensitivity to resistant cell lines than to sensitive ones, a phenomenon known as collateral sensitivity (CS). Interestingly, in this study *ABCB1/MDR1*-expressing resistant leukemia cell line showed greater sensitivity to nimbolide compared to sensitive cells. To best of our knowledge, this is the first time to report the potentiality of nimbolide to induce collateral sensitivity in P-glycoprotein-overexpressing cells. This finding suggests nimbolide as potent CS agent and supports its further development in this area. BCRP is another member of the ABC transporter-mediated classical MDR mechanism. The clinical relevance of BCRP as a transporter for broad-spectrum anticancer drugs with chemotherapy failure is well established [35]. Thus, agents that evade recognition and efflux by BCRP would be of great value. Our findings imply that BCRP may not play a role in resistance to nimbolide. In contrast, Both *ABCB5*-transfected and non-transfected HEK293 cells showed cross-resistance to nimbolide. Moreover, our results demonstrated that mutation of *TP53* or *EGFR* did not confer resistance towards nimbolide. EGFR was correlated with cancer progression and poor survival in addition to the development of resistance to cytotoxic agents [19]. *TP53* is the most commonly mutated gene in human cancer. Mutant *TP53* does not only lose its normal function as tumor suppressor and the maintenance of genome stability, but also gains oncogenic features, promotes malignant progression and mediates drug resistance. Cell death attenuation and its ability to exert anti-apoptotic effects towards chemotherapeutics were observed with mutant *TP53* [17]. Indeed, it trans-activates various death regulatory genes, and a high level of the *MDR1* gene expression was also correlated to mutant *TP53* status [36]. Nimbolide is supposed to bypass MDR mediated by the expression of *EGFR* and mutant *TP53*.

### Nimbolide-induced collateral sensitivity in P-glycoprotein-overexpressing cells

**Figure 7:**
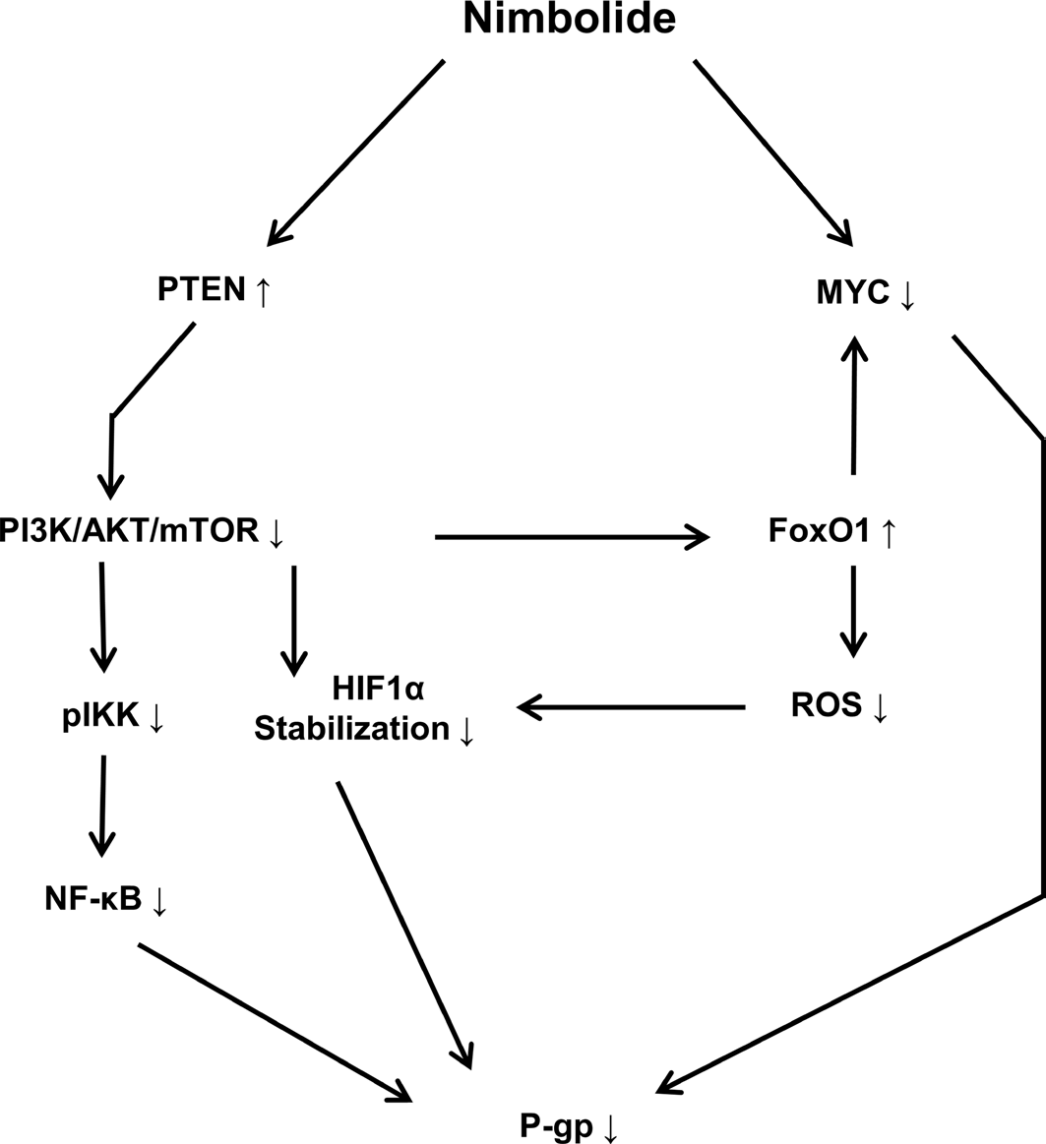
Schematic diagram showing the effect of nimbolide on P-glycoprotein expression and the involved molecular mediators Nimbolide-mediated P-glycoprotein downregulation is accomplished through targeting PTEN and MYC. PI3K/AKT/mTOR signaling pathway regulates NF-κB translocation and transcriptional activity through IKK phosphorylation. The inhibition of PI3K/AKT/mTOR leads to increasing HIF1α degradation and FoxO1 activation. The latter antagonizes MYC activity and decreases cellular ROS level.

The most remarkable observation from our cytotoxicity findings was the significant ability of nimbolide to inhibit cell viability of *ABCB1/MDR1* expressing cells compared to the sensitive parental cell line. In an attempt to understand the relevant genetic pathway and molecular networks that mediate collateral sensitivity, bioinformatical analyses were performed with the gene expression profiles of both cell lines. The results did not reveal significant differences affected by nimbolide with regard to main cellular functions, including cell death and survival, cellular development and cellular growth and proliferation. This is in good agreement with the reported multiple mechanisms that are influenced by nimbolide to exert its anticancer effect [37]. In contrast to CCRF–CEM cells, *MDR1*-expressing CEM/ADR5000 only highlighted alterations in cellular metabolism, such as nucleic acid metabolism, lipid metabolism, carbohydrate metabolism and free radical scavenging. Besides, the reduction in *ABCB1/MDR1* gene expression detected in microarray data, suggested that nimbolide might modulate P-glycoprotein expression at the transcriptional level. The upstream analysis highlighted significant PTEN activation and MYC inhibition. The tumor suppressor PTEN is well known as key negative regulator for the PI3K/Akt/mTOR pathway, and it has been proposed as powerful target for cancer treatment [38]. Several components in this signaling axis have been linked to anti-cancer drug resistance and most specifically to P-glycoprotein overexpression [39]. For instance, hypoxia inducible factor (HIF1α) is a well-known motif in the Akt/mTOR pathway with direct implications to affect P-glycoprotein expression and therapy resistance [40]. Our results showed a significant increase in the PTEN protein level and a reduction in HIF1α after nimbolide treatment. On the other hand, the redox systems also play a role for P-glycoprotein expression [41] and the ROS are well known to stabilize HIF1α [42]. In this context, our findings showed a reduction of cellular ROS after nimbolide treatment, suggested an indirect mechanism to regulate HIF1α through targeting ROS. It is worth to mention that high ROS production is proposed as one of the mechanisms for CS [34]. The hypothesis explains how P-glycoprotein substrates may stimulate ATPase activity and, therefore, why MDR cells preferentially die because of ROS generation and oxidative stress. Although nimbolide increased the cellular doxorubicin retention when we performed uptake assay to investigate its potentiality as P-glycoprotein substrate, we did not observe enhanced ROS production after treatment, which disprove the classical mechanism of CS. Indeed, nimbolide detoxifies the cellular baseline of ROS, downregulates MYC oncogene and upregulates FoxO1 transcription factor expressions, suggesting a novel mechanism for CS. MYC and FoxO are known in cancer metabolic adaptation and reprogramming, and the regulation of this axis is connected with the Akt/mTOR signaling pathway [43]. Taking together, we surmise that nimbolide induces CS in P-glycoprotein-overexpressing cells by targeting PTEN and modulating tumor cellular metabolic elements (HIF1α, FoxO1, c- MYC and ROS) (Figure 7).

### Analyses of microarray data using COMPARE and hierarchical cluster analysis

The COMPARE algorithm was used to identify compounds with similar growth inhibitory patterns as well as identifying molecular targets [44]. Genes from diverse different functional groups were identified as resistance and sensitivity mediators to nimbolide. Results were obtained from the correlation of mRNA expression profiles of the 60 cell lines panel in NCI with log_10_IC_50_ values of nimbolide. Regulators for DNA repair, replication and cell proliferation (*MSH5, RBBP4, HIST1H2AM, MCM7*) are example of genes found to mediate resistance to nimbolide. Other resistance genes exerted transcriptional activity (*NACA, TAF5*). In addition, *ANP32B* is one of the acidic (leucine-rich) nuclear phosphoprotein 32 kDa family members that has diverse physiological functions including apoptotic caspase modulation, transcriptional regulation, protein phosphorylation inhibition and regulation of intracellular transport [45]. The identification of *ANP32B* as resistance factor for nimbolide appears to be well substantiated by the study reported on *ANP32B* as negative regulator of apoptosis [46]. It was also suggested as tumor-promoting gene in breast cancer prognosis [45]. However, its causative relevance to mediate resistance towards nimbolide needs more investigations. Furthermore, it was not surprising that ribosomal proteins (*RPS15A, RPS21, RPL9, RPL36A, RPL17, RPL5, RPL34, RPS27*) and components of the mitochondrial respiratory chain (*UQCRH*) were identified as genes associated with resistance to nimbolide. Mediation of MDR by certain ribosomal proteins and their contribution toward cellular response has been reported as one of the extra-ribosomal functions [47]. Besides, the relation between the mitochondrial respiratory chain and chemotherapy resistance was previously described [48]. Via COMPARE analysis, another group of genes was linked to sensitivity towards nimbolide. Among them, *HIF1α* was the most interesting one. Especially, it has been marked up from our functional pathway analysis for collateral sensitivity-displayed cells as one of the targets. Consistent with our current work, a recent study has confirmed that nimbolide potentially targets HIFα [49]. For almost two decades, intensive research has been conducted on HIFs and HIF-related pathways due to their core involvement in therapy resistance and poor prognosis for patients [42].

Cluster analyses were performed as additional approach to predicted cellular responsiveness of the NCI 60 cell line panel by only including the mRNA expression profiles of the genes that were identified by COMPARE analysis. The IC_50_ values of the cell lines for nimbolide were not included in the cluster analysis. The genes were significantly distributed among five clusters, which emphasizes their potential relevance to mediate sensitivity and resistance to nimbolide.

### Inhibition of NF-κB by nimbolide

To obtain more in-depth insight of other molecular determinants that might contribute to cellular responses, we hypothesized that gene expression profiles identified by COMPARE analyses might be transcriptionally regulated by common transcription factors. Thus, the motif-screening strategy was applied to the genes’ upstream promoter sequences. Among the different transcription factors that appeared, NF-κB was significantly observed to have DNA binding motifs, indicating that NF-κB may play a considerable role in regulating those genes. On the other hand, literature evidence implies a direct role of NF-κB in tumor cell desensitization towards many chemotherapeutics and radiation therapy [22]. Constitutive activation of NF-κB has been reported in leukemia and many solid tumors (breast cancer, melanoma, colon cancer, and pancreatic cancer), associated with NK-κB-induced anti apoptotic effects [50]. NF-κB represents a potential molecular target, and searching for novel agents that inhibit its functions is strongly recommended [50]. This prompted us to investigate the effect of nimbolide towards NF-κB using reporter cell assay. We observed the possession of nimbolide to potent inhibitory effects, which supports previous findings in the literature [37]. Gupta *et al.* reported that nimbolide inhibited NF-κB pathway through inhibition of IKK activation, leading to suppression of NFKBIA phosphorylation, degradation and subsequent negative regulation of the expression of many tumorigenic proteins [51]. The phosphorylated and activated NF-κB is considered as one of the downstream targets of PI3K/Akt pathway, and inhibiting this transcription factor has been reported in many studies to decrease *ABCB1/MDR1* gene expression [52].

Our work has led us to conclude the potentiality of nimbolide to improve treatment success of tumors that especially express BCRP, P53 or EGFR multi-drug resistance mechanisms, as they do not play a role for nimbomide resistance. Strong collateral sensitivity towards P-glycoprotein (*ABCB1/MDR1*)-expressing cells was observed, and differential expression analysis suggested the involvement of essential cellular metabolic-regulating elements (HIF1α, FoxO1, MYC and ROS). Furthermore, nimbolide mediated collateral sensitivity through targeting PTEN and affecting its downstream components, resulting in significant downregulation of the *ABCB1/MDR1* gene and protein expression. Nimbolide possessed potent inhibitory effects against NF-κB activity and may thus enhance the effectiveness of antitumor therapy through sensitization of tumor cells to apoptosis induced by anticancer agents. Other molecular factors obtained from COMPARE analysis need further investigations for their relevant mechanisms of action. Interestingly, *HIF1α* was determined to mediate sensitivity to nimbolide, which would be of great benefit in targeted therapy.

## MATERIALS AND METHODS

### Human multidrug-resistant tumor cell lines

Parental, drug-sensitive CCRF–CEM leukemia cells and *MDR1*-expressing CEM/ADR5000 were cultured in RPMI 1640 medium (Invitrogen/Life Technologies, Darmstadt, Germany), supplemented with 10% fetal bovine serum (FBS) and 1% penicillin (1000 U/mL) and streptomycin (100 μg/mL) (Life Technologies) at standard conditions (humidified 5% CO_2_ atmosphere at 37° C). The maintenance of the resistance phenotype was accomplished by 5000 ng/ml doxorubicin once per week. The breast cancer cell lines MDA-MB-231 and MDA-MB-231-BCRP were maintained in DMEM medium (Invitrogen) supplemented with 10% FBS and 1% penicillin/streptomycin at standard conditions. Geneticin (800 ng/mL) (Invitrogen, Karlsruhe, Germany) was continuously added to the resistant subline to ensure the expression of breast cancer resistance protein (BCRP) [53]. HEK293 cells transfected with cDNA for *ABCB5* and non-transfected HEK293 cells, human glioblastoma multiforme U87.MGΔEGFR cells and parental U87.MG cell lines, in addition to human HCT116 p53^+/+^ wild-type colon cancer cells and HCT116 p53^−/−^ knockout cells were cultured under the same conditions as breast cancer cells. The inactivation of the *TP53* gene in HCT116 p53^−/−^ cells was generated by homologous recombination [54]. The resistant glioblastoma and colon cancer cell lines were maintained with 400 μg/mL geneticin. All cells were passaged twice weekly and experiments were performed with cells in the logarithmic growth phase.

### NCI 60 cell line panel

The Developmental Therapeutics Program (DTP) of the National Cancer Institute (NCI) cell line panel consists of 60 different human tumor, representing leukemia, melanoma and cancers of lung, colon, brain, ovary, breast, prostate, and kidney. The origin and processing of the cell lines have been previously described [55]. Nimbolide was tested against the NCI tumor panel.

### Cytotoxicity assay

Resazurin assay is based on the amount of viable cells that are able to convert the non-fluorescent indicator dye to a highly fluorescent one [56]. According to the previously described procedure [57], cells were cultured in a 96-well cell culture plate in a total volume of 200 μL, then treated with different concentrations of nimbolide. The concentration of DMSO was kept at or below 0.1%. After 72 h, 20 μL resazurin 0.01% w/v solution (Sigma-Aldrich, Schnelldorf, Germany) was added to each well, and the plates were incubated at 37° C for 3–4 h. Fluorescence was measured with the Infinite M2000 Pro™ plate reader (Tecan). Each assay was done at least three times, with six replicates each. The viability was compared based on a comparison with untreated cells. The concentration of sample required to inhibit 50% of cell proliferation (IC_50_ values) was calculated from a concentration-dependent curve by linear regression in Microsoft Excel.

### Gene expression profiling

Sensitive and resistant leukemia cell lines (CCRF–CEM and *MDR1*-expressing CEM/ADR5000) were subjected to total RNA extraction after 72 h of treatment with corresponding IC_50_ values of nimbolide or with DMSO solvent control. For this purpose, we used the RNeasy Kit from Qiagen (Hilden, Germany) and followed the manufacturer’s instructions. Microarray hybridizations were performed in duplicate for treated samples and for control samples by the Genomics and Proteomics Core Facility at the German Cancer Research Center (DKFZ, Heidelberg, Germany). The microarray hybridization procedure was previously described in detail [58].

### Validation of relative genes expression by quantitative reverse transcription PCR

According to RevertAid H Minus First Strand cDNA Synthesis kit instructions (Thermo Scientific, Darmstadt, Germany), 1 μg RNA template was converted to its complementary DNA, and directly used with 5× Hot Start Taq EvaGreen^®^ qPCR Mix (no ROX) (Axon Labortechnik, Kaiserslautern, Germany) for gene amplification. The PCR primer pairs were designed using NCBI/Primer-BLAST and obtained from Eurofins MWG Operon (Ebersberg, Germany) with the following sequences: *ABCB1* forward primer: ACCTGTGAAGAGTAGAACATGAAGA, *ABCB1* reverse primer: AATGTTCTGGCTTCCGTTGC, *MYC* forward primer: GTGGTCTTCCCCTACCCTCT, *MYC* reverse primer: GAGCAGAGAATCCGAGGACG, *MXD4* forward primer: TCACCACATGCTCCAACCTC, *MXD4* reverse primer: GGGCTCTGTTCTGCTTCTGT, *DDIT3* forward primer: CACCACACCTGAAAGCAGAT, *DDIT3* reverse primer: ATCTCTGCAGTTGGATCAGTC. QPCR was performed in CFX384™ (Bio-Rad, Munich, Germany) for 40 cycles. Each cycle includes, 95° C denaturation for 15 s, followed by 62–47° C gradient annealing step for 30 s, and 72° C elongation for 1 min at the end. The detailed analysis procedure for relative expression quantification is reported elsewhere [59].

### Doxorubicin uptake assay

Different concentrations of nimbolide (IC_50_, 2.5^*^IC_50_, 5^*^IC_50_) were used to treat cells (10^6^ cells/well in 6-well culture plates) incubated in transparent RPMI 1640 medium (without phenol red, Invitrogen™). The uptake of 20 μM doxorubicin (University Hospital Pharmacy, Mainz, Germany) was measured after 24 h in the presence or absence of nimbolide and compared to verapamil 100 μM, a known P-glycoprotein inhibitor (Sigma Aldrich, Taufkirchen, Germany). An excitation wavelength of 488 nm was selected, and doxorubicin mean fluorescence intensity (MFI) was measured using a band pass filter of 530/30 nm to collect the emitted light [60]. Measurements were performed by using a BD FACSCalibur™ (Beckton Dickinson, GmbH, Heidelberg, Germany), and the results were analyzed and visualized using FlowJo software.

### ROS quantification

Detection of reactive oxygen species (ROS) in multidrug-resistant CEM/ADR5000 leukemia cells was based on 2,7-dichlorodihydrofluorescein diacetate (H2DCFH-DA). The dye itself is non-fluorescent. It diffuses into the cells and the cytoplasmic esterase cleaves it to 2,7-dichlorodihydrofluorescein (H2DCF), which in turn oxidizes to a fluorescent molecule in the presence of ROS. Aliquots of 10^4^ cells/well were incubated in transparent RPMI 1640 medium for 3 h with different concentrations of nimbolide in 96-well plates. Afterwards, 10 μM dye was added 30 min prior the measurement and incubated in the dark. The fluorescent signals were recorded with an Infinite M2000 Pro™ plate reader (Tecan) at 495 nm excitation wavelength and 523 nm emission wavelength.

### Immunoblotting

Total protein was extracted from CEM/ADR5000 leukemia cells after treatment for 24 h with different concentrations of nimbolide, following the previously mentioned protocol [61]. Then, 8% SDS-PAGE and (Ruti^®^-PVDF) membrane (Millipore Corporation, Billerica, MA) were used for protein separation and blotting, respectively. After transferring proteins using a wet sandwich procedure, the membranes were blocked for 1 h with 5% (w\v) bovine serum album in T-TBS (Tris-buffered saline containing 0.5% Tween-20). Primary antibodies for P-glycoprotein, PTEN, HIF1α, FoxO1, MYC and β-actin (Cell Signaling Technology, Frankfurt, Germany), were diluted (1:1000) and incubated at 4° C with gentle shaking overnight. Membranes were washed thrice with T-TBS and incubated with anti-rabbit secondary antibody linked to horseradish peroxidase enzyme (1:2000) for 1 h at room temperature. Afterwards, Luminata™ Classico Western HRP substrate (Merck Millipore, Schwalbach, Germany) was added and incubated for 3 min in the dark. Blot signals were detected and analyzed with Alpha Innotech FluorChem Q system (Biozym, Oldendorf, Germany) [60].

### Bioinformatical methods

Chipster™ and Ingenuity pathway analysis (IPA)™ programs were applied to microarray data and used to analyze the change in genes expression levels in both leukemia cell lines after nimbolide treatment.

COMPARE algorithm developed by NCI (http://dtp.nci.nih.gov) was used to correlate nimbolide activity in terms of IC_50_ values with microarray-based mRNA expression profiles of the NCI panel of 60 cell lines. Since the COMPARE algorithm is based on Pearson’s correlation test, correlation coefficient (*R*-value) was obtained and ranked as a relative measure for the linear dependency of two variables [62]. Both standard and reverse COMPARE analyses were applied to identify genes that predict resistance (positive *R*-values) and sensitivity (negative *R*-values).

Hierarchical cluster analyses were performed to cluster the mRNA expression of genes identified by COMPARE analysis. A cluster tree or dendrogram was obtained by merging each individual object with another, depending on closeness of their characters using WinSTAT program (Kalmia Inc., Cambridge, MA). Then, the χ^2^-test was performed by taking the median of IC_50_ values of nimbolide as cut-off threshold [63].

Gene promoter analysis for transcription factor binding motifs was performed using Galaxy/Cistrome software available at (http://cistrome.org/ap/). Sequences of 40 genes that were associated with cellular response of tumor cells to nimbolide were changed to BED format using Table Browser in UCSC Genome Browser (https://genome.ucsc.edu/). Cistrome analysis platform was used to screen 25 kb upstream regions for all transcriptional factor-binding motifs via SeqPos tool [64].

### NF-κB reporter assay

HEK-Blue-Null1 cells were seeded in a density of (5 × 10^4^/mL) in 96-well plates and incubated overnight at 37° C. Cells were treated with 1 μM triptolide (Invivogen) (positive control) and different concentrations of nimbolide (1 μM, 0.1 μM, 0.01 μM and 0.001 μM) for 1 h. Then, 100 ng/mL TNF-α (Sigma-Aldrich) were added for 24 h to induce the inflammatory state in the cells. To detect NF-κB activation, 20 μL from the supernatant of cell culture were added to 180 μL of pre-warmed Quanti-Blue detection reagent (Invivogen) per well according to the manufacturer’s instructions. NF-κB activation was detected by measuring SEAP spectrophotometrically at 630 nm using a Tecan reader [64].

### Statistics

Results were obtained from three independent experiments and expressed as mean ± standard deviation (SD). Student’s *t*-test was used for statistical analysis, and *P* < 0.05 values were considered as statistically significant.

## SUPPLEMENTARY MATERIALS TABLES







## Abbreviations

ABC: ATP-binding cassette
BCRP: breast cancer resistance protein
CS: collateral sensitivity
EGFR: epidermal growth factor receptor
IPA: Ingenuity pathway analysis
MDR: multidrug resistance
NCI: National Cancer Institute
ROS: reactive oxygen species
TNF: tumor necrosis factor
TP53: tumor suppressor 53
