# Supplementary material for: Cytotoxicity of nimbolide towards multidrug-resistant tumor cells and hypersensitivity via cellular metabolic modulation
Source: Oncotarget. 2018 Nov 6;9(87):35762–79. doi: 10.18632/oncotarget.26299 (PMC6254660; doi:10.18632/oncotarget.26299)
Supplement: Supplementary file 1 [file oncotarget-09-35762-s001.pdf]

Cytotoxicity of nimbolide towards multidrug-resistant tumor cells and hypersensitivity via cellular metabolic modulation

SUPPLEMENTARY MATERIALS

Supplementary Table 1: The most significantly deregulated genes in CCRF-CEM cells after nimbolide treatment. See Supplementary_Table_1

Supplementary Table 2: The most significantly deregulated genes in CEM/ADR5000 cells after nimbolide treatment. See Supplementary_Table_2
