# Supplementary material for: Cytotoxicity of nimbolide towards multidrug-resistant tumor cells and hypersensitivity via cellular metabolic modulation
Source: Oncotarget. 2018 Nov 6;9(87):35762–79. doi: 10.18632/oncotarget.26299 (PMC6254660; doi:10.18632/oncotarget.26299)
Supplement: Supplementary file 2 [file oncotarget-09-35762-s002.docx]

**Supplementary Table 1.** The most significantly deregulated genes in CCRF-CEM cells after nimbolide

treatment.

| **Symbol** | **Description** | ***P*-value** | **FC** |
| --- | --- | --- | --- |
| *UBC* | ubiquitin C | 1.00E-06 | 3.170 |
| *FRG2B* | FSHD region gene 2 family member B | 2.00E-06 | 2.860 |
| *NR4A2* | nuclear receptor subfamily 4 group A member 2 | 1.00E-06 | 2.655 |
| *DLL1* | delta like canonical Notch ligand 1 | 1.00E-06 | 2.625 |
| *BCL6* | B cell CLL/lymphoma 6 | 1.00E-06 | 2.380 |
| *CHORDC1* | cysteine and histidine rich domain containing 1 | 1.00E-06 | 2.355 |
| *RAB6B* | RAB6B. member RAS oncogene family | 7.00E-06 | 2.345 |
| *CLK1* | CDC like kinase 1 | 6.00E-06 | 2.335 |
| *DDIT4* | DNA damage inducible transcript 4 | 1.00E-06 | 2.300 |
| *SNORD25* | small nucleolar RNA. C/D box 25 | 1.00E-06 | 2.295 |
| *HIST1H3A* | histone cluster 1 H3 family member a | 2.00E-06 | 2.290 |
| *ZC3H12A* | zinc finger CCCH-type containing 12A | 1.00E-06 | 2.270 |
| *PCDH10* | protocadherin 10 | 1.00E-06 | 2.215 |
| *SNORA28* | small nucleolar RNA. H/ACA box 28 | 1.00E-06 | 2.210 |
| *HIST1H1C* | histone cluster 1 H1 family member c | 1.00E-06 | 2.210 |
| *FAM46A* | family with sequence similarity 46 member A | 1.00E-06 | 2.125 |
| *MXD1* | MAX dimerization protein 1 | 1.00E-06 | 2.110 |
| *IFRD1* | interferon related developmental regulator 1 | 1.00E-06 | 2.110 |
| *HSP90AA1* | heat shock protein 90 alpha family class A member 1 | 1.00E-06 | 2.100 |
| *MIR425* | microRNA 425 | 2.00E-06 | 2.095 |
| *HIST1H3C* | histone cluster 1 H3 family member c | 1.00E-06 | 2.090 |
| *CCNE1* | cyclin E1 | 1.00E-06 | 2.080 |
| *TUFT1* | tuftelin 1 | 1.00E-06 | 2.080 |
| *SNORA63* | small nucleolar RNA. H/ACA box 63 | 1.00E-06 | 2.075 |
| *ATP1B1* | ATPase Na+/K+ transporting subunit beta 1 | 1.00E-06 | 2.075 |
| *DAND5* | DAN domain BMP antagonist family member 5 | 1.00E-06 | 2.065 |
| *CD69* | CD69 molecule | 1.00E-06 | 2.035 |
| *TSC22D3* | TSC22 domain family member 3 | 1.00E-06 | 2.035 |
| *EIF4A2P4* | eukaryotic translation initiation factor 4A2 pseudogene 4 | 1.00E-06 | 2.030 |
| *DUSP10* | dual specificity phosphatase 10 | 1.00E-05 | 2.010 |
| *SNORA16A* | small nucleolar RNA. H/ACA box 16A | 1.00E-06 | 2.010 |
| *EGR2* | early growth response 2 | 1.00E-06 | 2.010 |
| *HSPH1* | heat shock protein family H (Hsp110) member 1 | 1.00E-06 | 2.005 |
| *SNORD110* | small nucleolar RNA. C/D box 110 | 2.00E-06 | 2.005 |
| *RGS16* | regulator of G protein signaling 16 | 1.00E-06 | 1.990 |
| *ZFP36L1* | ZFP36 ring finger protein like 1 | 1.00E-06 | 1.985 |
| *CRISPLD2* | cysteine rich secretory protein LCCL domain containing 2 | 5.00E-06 | 1.985 |
| *MRPL18* | mitochondrial ribosomal protein L18 | 2.00E-06 | 1.970 |
| *CTH* | cystathionine gamma-lyase | 1.00E-06 | 1.965 |
| *ARRDC3* | arrestin domain containing 3 | 1.00E-06 | 1.960 |
| *HBG1* | hemoglobin subunit gamma 1 | 1.00E-06 | 1.960 |
| *SNAI1* | snail family transcriptional repressor 1 | 1.00E-06 | 1.945 |
| *LBH* | limb bud and heart development | 2.00E-06 | 1.945 |
| *CSRP2* | cysteine and glycine rich protein 2 | 2.00E-06 | 1.940 |
| *HBG2* | hemoglobin subunit gamma 2 | 1.00E-06 | 1.935 |
| *HIST2H2AB* | histone cluster 2 H2A family member b | 2.00E-06 | 1.935 |
| *ZFP36* | ZFP36 ring finger protein | 1.00E-06 | 1.930 |
| *KLF6* | Kruppel like factor 6 | 1.00E-06 | 1.915 |
| *ADAMTS1* | ADAM metallopeptidase with thrombospondin type 1 motif 1 | 1.70E-05 | 1.915 |
| *INSM2* | INSM transcriptional repressor 2 | 2.00E-06 | 1.915 |
| *SHISA2* | shisa family member 2 | 6.00E-06 | 1.915 |
| *PMAIP1* | phorbol-12-myristate-13-acetate-induced protein 1 | 2.00E-06 | 1.910 |
| *TSPYL2* | TSPY like 2 | 3.00E-06 | 1.910 |
| *RNF103* | ring finger protein 103 | 4.00E-06 | 1.910 |
| *HERPUD1* | homocysteine inducible ER protein with ubiquitin like domain 1 | 7.00E-06 | 1.895 |
| *TRIM51* | tripartite motif-containing 51 | 1.00E-06 | 1.885 |
| *RDH10* | retinol dehydrogenase 10 | 1.80E-05 | 1.870 |
| *KDM2A* | lysine demethylase 2A | 2.00E-06 | 1.860 |
| *CDO1* | cysteine dioxygenase type 1 | 4.00E-06 | 1.855 |
| *SNORD43* | small nucleolar RNA. C/D box 43 | 2.00E-06 | 1.850 |
| *C11orf96* | chromosome 11 open reading frame 96 | 3.00E-06 | 1.850 |
| *ARC* | activity regulated cytoskeleton associated protein | 1.00E-06 | 1.845 |
| *HIST1H2BF* | histone cluster 1 H2B family member f | 2.00E-06 | 1.845 |
| *NXT2* | nuclear transport factor 2 like export factor 2 | 9.00E-06 | 1.825 |
| *FXR1* | FMR1 autosomal homolog 1 | 3.00E-06 | 1.820 |
| *NFKBIA* | NFKB inhibitor alpha | 1.00E-06 | 1.815 |
| *SNORA67* | small nucleolar RNA. H/ACA box 67 | 2.00E-06 | 1.815 |
| *SNORA61* | small nucleolar RNA. H/ACA box 61 | 1.00E-06 | 1.810 |
| *CRYAB* | crystallin alpha B | 2.00E-06 | 1.810 |
| *HIST1H3J* | histone cluster 1 H3 family member j | 4.00E-06 | 1.810 |
| *METTL12* | citrate synthase lysine methyltransferase | 6.00E-06 | 1.810 |
| *IER2* | immediate early response 2 | 1.00E-06 | 1.805 |
| *TMEM2* | transmembrane protein 2 | 4.00E-06 | 1.800 |
| *INTS6* | integrator complex subunit 6 | 8.00E-06 | 1.800 |
| *FAM214A* | family with sequence similarity 214 member A | 2.00E-06 | 1.795 |
| *SNIP1* | Smad nuclear interacting protein 1 | 4.00E-06 | 1.775 |
| *RHOB* | ras homolog family member B | 2.00E-06 | 1.765 |
| *ACSL5* | acyl-CoA synthetase long chain family member 5 | 6.00E-06 | 1.765 |
| *PLA2G4C* | phospholipase A2 group IVC | 2.00E-06 | 1.760 |
| *DEDD2* | death effector domain containing 2 | 2.00E-06 | 1.750 |
| *NBPF10* | NBPF member 20 | 8.00E-06 | 1.745 |
| *RNU12* | RNA. U12 small nuclear | 2.00E-06 | 1.740 |
| *CD83* | CD83 molecule | 2.00E-06 | 1.730 |
| *UBE2C* | ubiquitin conjugating enzyme E2 C | 2.00E-06 | 1.720 |
| *RSRC2* | arginine and serine rich coiled-coil 2 | 2.00E-06 | 1.715 |
| *SNORA10* | small nucleolar RNA. H/ACA box 10 | 2.00E-06 | 1.700 |
| *TDRG1* | testis development related 1 | 3.00E-06 | 1.700 |
| *HIST1H3B* | histone cluster 1 H3 family member b | 3.00E-06 | 1.690 |
| *WDR47* | WD repeat domain 47 | 5.40E-05 | 1.685 |
| *EIF5* | eukaryotic translation initiation factor 5 | 1.90E-05 | 1.680 |
| *LAGE3P1* | L antigen family member 3 pseudogene 1 | 1.00E-05 | 1.665 |
| *TUBB2A* | tubulin beta 2A class IIa | 4.00E-06 | 1.665 |
| *DNAJB6* | DnaJ heat shock protein family (Hsp40) member B6 | 3.20E-05 | 1.655 |
| *MCL1* | MCL1. BCL2 family apoptosis regulator | 1.10E-05 | 1.645 |
| *CCDC173* | coiled-coil domain containing 173 | 1.30E-05 | 1.645 |
| *TIPARP* | TCDD inducible poly(ADP-ribose) polymerase | 7.00E-06 | 1.645 |
| *CA2* | carbonic anhydrase 2 | 7.00E-06 | 1.645 |
| *AHSA2* | activator of HSP90 ATPase homolog 2. pseudogene | 3.00E-06 | 1.640 |
| *LINC00936* | ATP2B1 antisense RNA 1 | 3.00E-06 | 1.640 |
| *TAF7* | TATA-box binding protein associated factor 7 | 1.50E-05 | 1.625 |
| *HSP90AB1* | heat shock protein 90 alpha family class B member 1 | 7.00E-06 | 1.620 |
| *HSPB1* | heat shock protein family B (small) member 1 | 3.00E-06 | 1.615 |
| *MB21D1* | cyclic GMP-AMP synthase | 6.60E-05 | 1.615 |
| *CCDC121* | coiled-coil domain containing 121 | 1.30E-05 | 1.610 |
| *HSPA4L* | heat shock protein family A (Hsp70) member 4 like | 2.50E-05 | 1.605 |
| *TOB1* | transducer of ERBB2. 1 | 2.00E-05 | 1.600 |
| *FAM90A15P* | family with sequence similarity 90 member A26 | 4.00E-06 | 1.600 |
| *CCNL1* | cyclin L1 | 5.00E-06 | 1.600 |
| *CRYBA4* | crystallin beta A4 | 5.00E-06 | 1.600 |
| *SNORD68* | small nucleolar RNA. C/D box 68 | 6.00E-06 | 1.595 |
| *ACTA1* | actin. alpha 1. skeletal muscle | 1.10E-05 | 1.590 |
| *EFNB2* | ephrin B2 | 7.00E-06 | 1.580 |
| *MEX3B* | mex-3 RNA binding family member B | 2.90E-05 | 1.575 |
| *ZNF493* | zinc finger protein 493 | 3.00E-06 | 1.575 |
| *HIST1H2BE* | histone cluster 1 H2B family member e | 3.00E-06 | 1.570 |
| *ZNF622* | zinc finger protein 622 | 3.00E-06 | 1.570 |
| *ZNF684* | zinc finger protein 684 | 4.00E-06 | 1.570 |
| *IDH1* | isocitrate dehydrogenase (NADP(+)) 1. cytosolic | 4.00E-06 | 1.565 |
| *SNORA80A* | small nucleolar RNA. H/ACA box 80A | 7.00E-06 | 1.565 |
| *H2AFJ* | H2A histone family member J | 3.00E-06 | 1.560 |
| *MYCN* | MYCN proto-oncogene. bHLH transcription factor | 1.20E-05 | 1.550 |
| *HSPA2* | heat shock protein family A (Hsp70) member 2 | 1.20E-05 | 1.545 |
| *RWDD1* | RWD domain containing 1 | 3.00E-06 | 1.545 |
| *SNORD57* | small nucleolar RNA. C/D box 57 | 1.00E-05 | 1.540 |
| *FAM83D* | family with sequence similarity 83 member D | 4.00E-06 | 1.540 |
| *WBP5* | transcription elongation factor A like 9 | 6.00E-06 | 1.530 |
| *SAT1* | spermidine/spermine N1-acetyltransferase 1 | 4.00E-06 | 1.520 |
| *RPL10L* | ribosomal protein L10 like | 5.00E-06 | 1.510 |
| *TSC22D1* | TSC22 domain family member 1 | 6.00E-06 | 1.505 |
| *SIRT4* | sirtuin 4 | 1.00E-05 | 1.495 |
| *CDH3* | cadherin 3 | 1.60E-05 | 1.495 |
| *PPP1R10* | protein phosphatase 1 regulatory subunit 10 | 4.00E-06 | 1.495 |
| *LSMEM1* | leucine rich single-pass membrane protein 1 | 1.40E-05 | 1.490 |
| *CALM1* | calmodulin 1 | 6.00E-06 | 1.490 |
| *KLF2* | Kruppel like factor 2 | 8.00E-06 | 1.490 |
| *ZNF844* | zinc finger protein 844 | 2.10E-05 | 1.485 |
| *MIRLET7I* | microRNA let-7i | 1.30E-05 | 1.480 |
| *ZNF20* | zinc finger protein 20 | 8.00E-06 | 1.480 |
| *ZSWIM3* | zinc finger SWIM-type containing 3 | 8.00E-06 | 1.470 |
| *C2orf42* | chromosome 2 open reading frame 42 | 1.30E-05 | 1.460 |
| *SCG5* | secretogranin V | 1.60E-05 | 1.445 |
| *AHSA1* | activator of HSP90 ATPase activity 1 | 8.00E-06 | 1.440 |
| *UGP2* | UDP-glucose pyrophosphorylase 2 | 1.00E-05 | 1.435 |
| *SNORD16* | small nucleolar RNA. C/D box 16 | 1.10E-05 | 1.435 |
| *SNHG17* | small nucleolar RNA host gene 17 | 8.00E-06 | 1.435 |
| *PHLDA1* | pleckstrin homology like domain family A member 1 | 6.00E-06 | 1.430 |
| *CDK7* | cyclin dependent kinase 7 | 9.00E-06 | 1.430 |
| *HIST1H3D* | histone cluster 1 H3 family member d | 1.00E-05 | 1.425 |
| *PRRG2* | proline rich and Gla domain 2 | 2.10E-05 | 1.425 |
| *NRDE2* | NRDE-2. necessary for RNA interference. domain containing | 6.00E-06 | 1.425 |
| *TMEM223* | transmembrane protein 223 | 8.00E-06 | 1.425 |
| *SNORA7B* | small nucleolar RNA. H/ACA box 7B | 2.40E-05 | 1.420 |
| *MYBL1* | MYB proto-oncogene like 1 | 9.00E-06 | 1.420 |
| *FOXC1* | forkhead box C1 | 1.20E-05 | 1.415 |
| *RND1* | Rho family GTPase 1 | 1.40E-05 | 1.415 |
| *RYBP* | RING1 and YY1 binding protein | 3.10E-05 | 1.410 |
| *EIF4A2* | eukaryotic translation initiation factor 4A2 | 6.00E-06 | 1.410 |
| *NUDT9* | nudix hydrolase 9 | 8.00E-06 | 1.410 |
| *ENPP2* | ectonucleotide pyrophosphatase/phosphodiesterase 2 | 1.10E-05 | 1.405 |
| *SNORD15B* | small nucleolar RNA. C/D box 15B | 3.20E-05 | 1.405 |
| *GBP2* | guanylate binding protein 2 | 2.90E-05 | 1.400 |
| *EPB41L4A-AS1* | EPB41L4A antisense RNA 1 | 6.00E-06 | 1.400 |
| *CDKN2C* | cyclin dependent kinase inhibitor 2C | 1.20E-05 | 1.390 |
| *BMP4* | bone morphogenetic protein 4 | 1.20E-05 | 1.385 |
| *HIST1H4I* | histone cluster 1 H4 family member i | 1.70E-05 | 1.385 |
| *ZNF211* | zinc finger protein 211 | 1.30E-05 | 1.380 |
| *GSKIP* | GSK3B interacting protein | 2.50E-05 | 1.380 |
| *KLF10* | Kruppel like factor 10 | 1.00E-05 | 1.370 |
| *GLB1L* | galactosidase beta 1 like | 1.80E-05 | 1.355 |
| *ZNF34* | zinc finger protein 34 | 2.30E-05 | 1.355 |
| *CTSV* | cathepsin V | 1.00E-05 | 1.350 |
| *BPGM* | bisphosphoglycerate mutase | 1.40E-05 | 1.350 |
| *SREK1IP1* | SREK1 interacting protein 1 | 2.50E-05 | 1.350 |
| *HES6* | hes family bHLH transcription factor 6 | 9.00E-06 | 1.340 |
| *MSMO1* | methylsterol monooxygenase 1 | 1.30E-05 | 1.335 |
| *RPL13P5* | ribosomal protein L13 pseudogene 5 | 4.70E-05 | 1.335 |
| *SNORD30* | small nucleolar RNA. C/D box 30 | 1.00E-05 | 1.330 |
| *S100P* | S100 calcium binding protein P | 1.00E-05 | 1.330 |
| *WDR19* | WD repeat domain 19 | 1.30E-05 | 1.330 |
| *HNRNPDL* | heterogeneous nuclear ribonucleoprotein D like | 1.30E-05 | 1.330 |
| *LRIF1* | ligand dependent nuclear receptor interacting factor 1 | 9.00E-05 | 1.330 |
| *ASNS* | asparagine synthetase (glutamine-hydrolyzing) | 1.30E-05 | 1.325 |
| *SLC7A5P1* | solute carrier family 7 member 5 pseudogene 1 | 1.40E-05 | 1.325 |
| *PTGER4* | prostaglandin E receptor 4 | 2.10E-05 | 1.325 |
| *KLF4* | Kruppel like factor 4 | 2.30E-05 | 1.325 |
| *FHL2* | four and a half LIM domains 2 | 1.50E-05 | 1.320 |
| *HIST1H2AE* | histone cluster 1 H2A family member e | 1.20E-05 | 1.315 |
| *AP4B1* | adaptor related protein complex 4 beta 1 subunit | 1.00E-05 | 1.310 |
| *TAF1D* | TATA-box binding protein associated factor. RNA polymerase I subunit D | 1.60E-05 | 1.305 |
| *ZFAS1* | ZNFX1 antisense RNA 1 | 1.70E-05 | 1.305 |
| *ANKRD36B* | ankyrin repeat domain 36B | 7.90E-05 | 1.300 |
| *OSER1* | oxidative stress responsive serine rich 1 | 1.20E-05 | 1.295 |
| *TRIM53CP* | tripartite motif containing 53A. pseudogene | 1.30E-05 | 1.280 |
| *CCNG2* | cyclin G2 | 4.40E-05 | 1.280 |
| *HSD17B7P2* | hydroxysteroid 17-beta dehydrogenase 7 pseudogene 2 | 1.50E-05 | 1.275 |
| *ATF4* | activating transcription factor 4 | 1.60E-05 | 1.275 |
| *ATG16L1* | autophagy related 16 like 1 | 2.40E-05 | 1.275 |
| *PPM1D* | protein phosphatase. Mg2+/Mn2+ dependent 1D | 2.70E-05 | 1.275 |
| *ETNK1* | ethanolamine kinase 1 | 1.30E-05 | 1.270 |
| *VASN* | vasorin | 2.70E-05 | 1.270 |
| *PSPC1* | paraspeckle component 1 | 1.80E-05 | 1.260 |
| *ENO3* | enolase 3 | 2.30E-05 | 1.260 |
| *PNMAL1* | PNMA family member 8A | 5.20E-05 | 1.260 |
| *SERTAD3* | SERTA domain containing 3 | 6.10E-05 | 1.260 |
| *ZRSR2* | zinc finger CCCH-type. RNA binding motif and serine/arginine rich 2 | 1.90E-05 | 1.255 |
| *TMEM31* | transmembrane protein 31 | 3.10E-05 | 1.255 |
| *ABHD3* | abhydrolase domain containing 3 | 3.20E-05 | 1.250 |
| *VTRNA2-1* | vault RNA 2-1 | 1.50E-05 | 1.245 |
| *RBM23* | RNA binding motif protein 23 | 1.50E-05 | 1.245 |
| *VCPKMT* | valosin containing protein lysine methyltransferase | 2.10E-05 | 1.240 |
| *TUBA4A* | tubulin alpha 4a | 1.70E-05 | 1.235 |
| *IER5L* | immediate early response 5 like | 1.40E-05 | 1.230 |
| *DPF3* | double PHD fingers 3 | 1.50E-05 | 1.230 |
| *ZNF256* | zinc finger protein 256 | 1.70E-05 | 1.230 |
| *FAM53C* | family with sequence similarity 53 member C | 2.20E-05 | 1.230 |
| *SERPINE1* | serpin family E member 1 | 3.45E-04 | 1.230 |
| *NFAT5* | nuclear factor of activated T cells 5 | 4.80E-05 | 1.230 |
| *SIK1* | salt inducible kinase 1 | 1.40E-05 | 1.225 |
| *RBM4B* | RNA binding motif protein 4B | 1.40E-05 | 1.225 |
| *HEY1* | hes related family bHLH transcription factor with YRPW motif 1 | 1.90E-05 | 1.225 |
| *HES1* | hes family bHLH transcription factor 1 | 1.70E-05 | 1.220 |
| *SNORD83B* | small nucleolar RNA. C/D box 83B | 1.80E-05 | 1.220 |
| *SMG8* | SMG8. nonsense mediated mRNA decay factor | 2.00E-05 | 1.205 |
| *ARID4A* | AT-rich interaction domain 4A | 2.46E-04 | 1.205 |
| *ABHD5* | abhydrolase domain containing 5 | 3.60E-05 | 1.205 |
| *PAPD5* | poly(A) RNA polymerase D5. non-canonical | 1.00E-04 | 1.200 |
| *ZIK1* | zinc finger protein interacting with K protein 1 | 4.30E-05 | 1.200 |
| *FKBP14* | FK506 binding protein 14 | 4.90E-05 | 1.200 |
| *PNP* | purine nucleoside phosphorylase | 1.50E-05 | 1.195 |
| *CEBPB* | CCAAT/enhancer binding protein beta | 1.90E-05 | 1.195 |
| *SNORD95* | small nucleolar RNA. C/D box 95 | 2.20E-05 | 1.195 |
| *TPM2* | tropomyosin 2 | 1.50E-05 | 1.190 |
| *SNORA14A* | small nucleolar RNA. H/ACA box 14A | 3.20E-05 | 1.190 |
| *ANKMY2* | ankyrin repeat and MYND domain containing 2 | 5.70E-05 | 1.190 |
| *MPHOSPH8* | M-phase phosphoprotein 8 | 1.60E-05 | 1.185 |
| *SLC25A3* | solute carrier family 25 member 3 | 2.00E-05 | 1.185 |
| *SLC7A5P2* | solute carrier family 7 member 5 pseudogene 2 | 4.20E-05 | 1.185 |
| *KLHL15* | kelch like family member 15 | 9.10E-05 | 1.185 |
| *ZNF439* | zinc finger protein 439 | 2.00E-05 | 1.180 |
| *HIST1H2BC* | histone cluster 1 H2B family member c | 3.40E-05 | 1.180 |
| *RAE1* | ribonucleic acid export 1 | 2.50E-05 | 1.175 |
| *AGAP6* | ArfGAP with GTPase domain. ankyrin repeat and PH domain 5 | 3.20E-05 | 1.175 |
| *MIR1292* | microRNA 1292 | 2.10E-05 | 1.160 |
| *OVGP1* | oviductal glycoprotein 1 | 2.20E-05 | 1.155 |
| *SYT11* | synaptotagmin 11 | 3.90E-05 | 1.155 |
| *RBM5* | RNA binding motif protein 5 | 2.50E-05 | 1.150 |
| *SNORD74* | small nucleolar RNA. C/D box 74 | 2.00E-05 | 1.145 |
| *CTBP2P5* | C-terminal binding protein 2 pseudogene 5 | 2.40E-05 | 1.145 |
| *ANKRD36* | ankyrin repeat domain 36 | 2.31E-04 | 1.140 |
| *DHRS2* | dehydrogenase/reductase 2 | 2.68E-04 | 1.140 |
| *SNHG7* | small nucleolar RNA host gene 7 | 3.00E-05 | 1.140 |
| *ZNF816* | zinc finger protein 816 | 3.10E-05 | 1.140 |
| *SNORA76C* | small nucleolar RNA. H/ACA box 50C | 2.60E-05 | 1.135 |
| *RBM39* | RNA binding motif protein 39 | 1.40E-04 | 1.130 |
| *DCUN1D3* | defective in cullin neddylation 1 domain containing 3 | 2.00E-05 | 1.130 |
| *NPAS4* | neuronal PAS domain protein 4 | 2.70E-05 | 1.130 |
| *SETX* | senataxin | 1.15E-04 | 1.125 |
| *CTR9* | CTR9 homolog. Paf1/RNA polymerase II complex component | 3.01E-04 | 1.125 |
| *SLCO4C1* | solute carrier organic anion transporter family member 4C1 | 3.50E-05 | 1.120 |
| *FAM24B* | family with sequence similarity 24 member B | 2.60E-05 | 1.115 |
| *FAM179B* | TOG array regulator of axonemal microtubules 1 | 5.10E-05 | 1.115 |
| *EDRF1* | erythroid differentiation regulatory factor 1 | 7.70E-05 | 1.115 |
| *SLC30A1* | solute carrier family 30 member 1 | 8.78E-04 | 1.115 |
| *ZKSCAN4* | zinc finger with KRAB and SCAN domains 4 | 1.01E-04 | 1.110 |
| *WHAMM* | WAS protein homolog associated with actin. golgi membranes and microtubules | 2.68E-04 | 1.110 |
| *EED* | embryonic ectoderm development | 3.70E-05 | 1.110 |
| *HIST1H2AH* | histone cluster 1 H2A family member h | 3.80E-05 | 1.110 |
| *YTHDC1* | YTH domain containing 1 | 9.80E-05 | 1.110 |
| *MOK* | MOK protein kinase | 4.40E-05 | 1.105 |
| *IQCB1* | IQ motif containing B1 | 3.00E-05 | 1.100 |
| *TYW1B* | tRNA-yW synthesizing protein 1 homolog B | 3.90E-05 | 1.100 |
| *VAMP1* | vesicle associated membrane protein 1 | 3.40E-05 | 1.095 |
| *MKRN3* | makorin ring finger protein 3 | 3.80E-05 | 1.095 |
| *MSL3* | MSL complex subunit 3 | 6.60E-05 | 1.095 |
| *SIRT1* | sirtuin 1 | 3.10E-05 | 1.090 |
| *FAM133B* | family with sequence similarity 133 member B | 3.80E-05 | 1.090 |
| *ASTE1* | asteroid homolog 1 | 3.00E-05 | 1.085 |
| *LIMA1* | LIM domain and actin binding 1 | 3.60E-05 | 1.085 |
| *NEUROG2* | neurogenin 2 | 1.12E-04 | 1.080 |
| *BEX1* | brain expressed X-linked 1 | 3.50E-05 | 1.080 |
| *TUBB3* | tubulin beta 3 class III | 2.90E-05 | 1.075 |
| *INTS12* | integrator complex subunit 12 | 3.60E-05 | 1.075 |
| *KDM3A* | lysine demethylase 3A | 5.40E-05 | 1.075 |
| *CCDC77* | coiled-coil domain containing 77 | 5.70E-05 | 1.075 |
| *JUNB* | JunB proto-oncogene. AP-1 transcription factor subunit | 1.83E-04 | 1.065 |
| *ZNF682* | zinc finger protein 682 | 3.21E-04 | 1.065 |
| *ARIH2OS* | ariadne RBR E3 ubiquitin protein ligase 2 opposite strand | 1.07E-04 | 1.060 |
| *ZBTB43* | zinc finger and BTB domain containing 43 | 1.40E-04 | 1.060 |
| *MKNK2* | MAP kinase interacting serine/threonine kinase 2 | 1.16E-04 | 1.055 |
| *RIOK3* | RIO kinase 3 | 2.87E-04 | 1.055 |
| *TUBB7P* | tubulin beta 7 pseudogene | 3.40E-05 | 1.055 |
| *HLA-DRB4* | major histocompatibility complex. class II. DR beta 4 | 4.20E-05 | 1.055 |
| *ANKRD20A11P* | ankyrin repeat domain 20 family member A11. pseudogene | 2.49E-04 | 1.045 |
| *NPIPB6* | nuclear pore complex interacting protein family member B8 | 3.40E-05 | 1.045 |
| *BRD2* | bromodomain containing 2 | 3.40E-05 | 1.045 |
| *HIST1H2BN* | histone cluster 1 H2B family member n | 3.40E-05 | 1.045 |
| *KIAA1468* | KIAA1468 | 4.40E-05 | 1.045 |
| *ARID4B* | AT-rich interaction domain 4B | 2.35E-03 | 1.040 |
| *SNORD52* | small nucleolar RNA. C/D box 52 | 4.10E-05 | 1.040 |
| *TUBE1* | tubulin epsilon 1 | 4.90E-05 | 1.040 |
| *FRAT2* | FRAT2. WNT signaling pathway regulator | 3.80E-05 | 1.035 |
| *SNORD84* | small nucleolar RNA. C/D box 84 | 6.40E-05 | 1.035 |
| *FBXO30* | F-box protein 30 | 6.80E-05 | 1.035 |
| *SLC5A3* | solute carrier family 5 member 3 | 2.68E-04 | 1.030 |
| *GNL3* | G protein nucleolar 3 | 3.50E-05 | 1.030 |
| *ZC3H11A* | zinc finger CCCH-type containing 11A | 1.40E-04 | 1.025 |
| *KIAA0895* | KIAA0895 | 2.01E-04 | 1.025 |
| *ZNF669* | zinc finger protein 669 | 4.20E-05 | 1.025 |
| *UBXN8* | UBX domain protein 8 | 7.90E-05 | 1.025 |
| *FAM46C* | family with sequence similarity 46 member C | 8.00E-05 | 1.025 |
| *HBP1* | HMG-box transcription factor 1 | 9.40E-05 | 1.025 |
| *SLC3A2* | solute carrier family 3 member 2 | 1.11E-04 | 1.020 |
| *SNORA33* | small nucleolar RNA. H/ACA box 33 | 4.50E-05 | 1.020 |
| *BTG2* | BTG anti-proliferation factor 2 | 3.80E-05 | 1.015 |
| *TP53BP1* | tumor protein p53 binding protein 1 | 8.00E-05 | 1.015 |
| *EFNB3* | ephrin B3 | 3.83E-04 | 1.010 |
| *ING3* | inhibitor of growth family member 3 | 1.40E-04 | 1.005 |
| *ZCCHC8* | zinc finger CCHC-type containing 8 | 2.94E-04 | 1.005 |
| *IRX5* | iroquois homeobox 5 | 3.80E-05 | 1.005 |
| *MRFAP1L1* | Morf4 family associated protein 1 like 1 | 4.20E-05 | 1.005 |
| *SOD2* | superoxide dismutase 2 | 4.60E-05 | 1.005 |
| *NEK2* | NIMA related kinase 2 | 4.70E-05 | 1.005 |
| *SNORD35A* | small nucleolar RNA. C/D box 35A | 5.20E-05 | 1.005 |
| *RHEBL1* | RHEB like 1 | 6.30E-05 | 1.005 |
| *ATP6V1B1* | ATPase H+ transporting V1 subunit B1 | 8.80E-05 | 1.005 |
| *HYPK* | huntingtin interacting protein K | 1.06E-03 | 1.000 |
| *STARD5* | StAR related lipid transfer domain containing 5 | 4.70E-05 | 1.000 |
| *SNORA24* | small nucleolar RNA. H/ACA box 24 | 5.40E-05 | 1.000 |
| *ZNF511* | zinc finger protein 511 | 2.15E-04 | -1.000 |
| *PDXK* | pyridoxal kinase | 4.10E-05 | -1.000 |
| *DPY19L1* | dpy-19 like C-mannosyltransferase 1 | 4.30E-05 | -1.000 |
| *RAG1* | recombination activating 1 | 5.29E-04 | -1.000 |
| *TMED7* | transmembrane p24 trafficking protein 7 | 6.50E-05 | -1.000 |
| *TSKU* | tsukushi. small leucine rich proteoglycan | 7.40E-05 | -1.000 |
| *ST8SIA4* | ST8 alpha-N-acetyl-neuraminide alpha-2.8-sialyltransferase 4 | 4.40E-05 | -1.005 |
| *PTPN11* | protein tyrosine phosphatase. non-receptor type 11 | 4.60E-05 | -1.005 |
| *STK40* | serine/threonine kinase 40 | 6.30E-05 | -1.005 |
| *ELK1* | ELK1. ETS transcription factor | 7.90E-05 | -1.005 |
| *NDUFA9* | NADH:ubiquinone oxidoreductase subunit A9 | 9.10E-05 | -1.005 |
| *POC1A* | POC1 centriolar protein A | 1.27E-04 | -1.010 |
| *BYSL* | bystin like | 5.81E-04 | -1.010 |
| *CD1C* | CD1c molecule | 7.40E-05 | -1.010 |
| *RAB40C* | RAB40C. member RAS oncogene family | 2.25E-04 | -1.015 |
| *TMED1* | transmembrane p24 trafficking protein 1 | 2.29E-04 | -1.015 |
| *MRPL14* | mitochondrial ribosomal protein L14 | 3.15E-04 | -1.015 |
| *MFSD5* | major facilitator superfamily domain containing 5 | 3.80E-05 | -1.015 |
| *UTP11L* | UTP11. small subunit processome component | 4.70E-05 | -1.015 |
| *ARPP19* | cAMP regulated phosphoprotein 19 | 5.10E-05 | -1.015 |
| *MSRB1* | methionine sulfoxide reductase B1 | 5.28E-04 | -1.015 |
| *ASB8* | ankyrin repeat and SOCS box containing 8 | 7.40E-05 | -1.015 |
| *ZBTB42* | zinc finger and BTB domain containing 42 | 9.50E-05 | -1.015 |
| *HPS6* | HPS6. biogenesis of lysosomal organelles complex 2 subunit 3 | 1.07E-04 | -1.020 |
| *FBLN2* | fibulin 2 | 1.19E-04 | -1.020 |
| *NINJ2* | ninjurin 2 | 1.74E-04 | -1.020 |
| *ACTN1* | actinin alpha 1 | 3.80E-05 | -1.020 |
| *DCP2* | decapping mRNA 2 | 3.80E-05 | -1.020 |
| *ABCD3* | ATP binding cassette subfamily D member 3 | 3.50E-05 | -1.025 |
| *CHI3L2* | chitinase 3 like 2 | 3.90E-05 | -1.025 |
| *TOX* | thymocyte selection associated high mobility group box | 5.20E-05 | -1.025 |
| *NXT1* | nuclear transport factor 2 like export factor 1 | 6.20E-05 | -1.025 |
| *PPP1CB* | protein phosphatase 1 catalytic subunit beta | 8.20E-05 | -1.025 |
| *LAMTOR1* | late endosomal/lysosomal adaptor. MAPK and MTOR activator 1 | 9.80E-05 | -1.030 |
| *PAQR4* | progestin and adipoQ receptor family member 4 | 3.60E-05 | -1.035 |
| *GID8* | GID complex subunit 8 homolog | 4.10E-05 | -1.035 |
| *IGLL1* | immunoglobulin lambda like polypeptide 5 | 5.10E-05 | -1.035 |
| *VAMP8* | vesicle associated membrane protein 8 | 1.51E-04 | -1.040 |
| *FAF2* | Fas associated factor family member 2 | 3.20E-05 | -1.040 |
| *AHCTF1* | AT-hook containing transcription factor 1 | 3.70E-05 | -1.040 |
| *E2F3* | E2F transcription factor 3 | 3.80E-05 | -1.040 |
| *WIPF1* | WAS/WASL interacting protein family member 1 | 3.90E-05 | -1.040 |
| *DBNL* | drebrin like | 9.90E-05 | -1.040 |
| *CBL* | Cbl proto-oncogene | 3.90E-05 | -1.045 |
| *PUDP* | pseudouridine 5'-phosphatase | 4.20E-05 | -1.045 |
| *PTBP3* | polypyrimidine tract binding protein 3 | 4.20E-05 | -1.045 |
| *GTPBP4* | GTP binding protein 4 | 5.20E-05 | -1.045 |
| *USP9X* | ubiquitin specific peptidase 9. X-linked | 3.80E-05 | -1.050 |
| *MME* | membrane metalloendopeptidase | 4.60E-05 | -1.050 |
| *METTL1* | methyltransferase like 1 | 5.40E-05 | -1.050 |
| *MAP6D1* | MAP6 domain containing 1 | 5.60E-05 | -1.050 |
| *EIF4EP2* | eukaryotic translation initiation factor 4E pseudogene 2 | 6.20E-05 | -1.050 |
| *CYB561D2* | cytochrome b561 family member D2 | 8.60E-05 | -1.050 |
| *HNRNPAB* | heterogeneous nuclear ribonucleoprotein A/B | 1.07E-04 | -1.055 |
| *CNPY2* | canopy FGF signaling regulator 2 | 2.50E-04 | -1.055 |
| *PAK2* | p21 (RAC1) activated kinase 2 | 3.00E-05 | -1.055 |
| *API5* | apoptosis inhibitor 5 | 3.20E-05 | -1.060 |
| *POLR3H* | RNA polymerase III subunit H | 3.60E-05 | -1.070 |
| *CERS6* | ceramide synthase 6 | 4.10E-05 | -1.070 |
| *MRPL27* | mitochondrial ribosomal protein L27 | 5.26E-04 | -1.070 |
| *PPIA* | peptidylprolyl isomerase A | 3.50E-05 | -1.075 |
| *CD79B* | CD79b molecule | 4.80E-05 | -1.075 |
| *LIN28B* | lin-28 homolog B | 5.20E-05 | -1.075 |
| *SRSF1* | serine and arginine rich splicing factor 1 | 2.60E-05 | -1.080 |
| *SCD* | stearoyl-CoA desaturase | 2.70E-05 | -1.080 |
| *NOTCH3* | notch 3 | 2.80E-05 | -1.080 |
| *MCM10* | minichromosome maintenance 10 replication initiation factor | 3.50E-05 | -1.080 |
| *AIF1L* | allograft inflammatory factor 1 like | 3.90E-05 | -1.080 |
| *FEN1* | flap structure-specific endonuclease 1 | 4.50E-05 | -1.080 |
| *NKX2-5* | NK2 homeobox 5 | 5.30E-05 | -1.080 |
| *ZBTB21* | zinc finger and BTB domain containing 21 | 6.00E-05 | -1.080 |
| *CALU* | calumenin | 3.40E-05 | -1.085 |
| *ELOVL6* | ELOVL fatty acid elongase 6 | 4.90E-05 | -1.085 |
| *CARHSP1* | calcium regulated heat stable protein 1 | 1.52E-04 | -1.090 |
| *TMEM170B* | transmembrane protein 170B | 4.70E-05 | -1.090 |
| *PXDN* | peroxidasin | 7.90E-05 | -1.090 |
| *TMEM30A* | transmembrane protein 30A | 2.50E-05 | -1.095 |
| *PTPRF* | protein tyrosine phosphatase. receptor type F | 7.20E-05 | -1.100 |
| *MIEF1* | mitochondrial elongation factor 1 | 2.50E-05 | -1.105 |
| *LINC00116* | small integral membrane protein 37 | 3.50E-05 | -1.105 |
| *WDR18* | WD repeat domain 18 | 4.20E-05 | -1.110 |
| *B4GALT6* | beta-1.4-galactosyltransferase 6 | 2.50E-05 | -1.115 |
| *S100A4* | S100 calcium binding protein A4 | 4.90E-05 | -1.115 |
| *SAMM50* | SAMM50 sorting and assembly machinery component | 4.30E-05 | -1.125 |
| *BCL2* | BCL2. apoptosis regulator | 2.50E-05 | -1.130 |
| *WRB* | tryptophan rich basic protein | 2.96E-04 | -1.130 |
| *CD47* | CD47 molecule | 2.10E-05 | -1.135 |
| *LBR* | lamin B receptor | 4.00E-05 | -1.135 |
| *SERBP1* | SERPINE1 mRNA binding protein 1 | 4.20E-05 | -1.135 |
| *PXMP2* | peroxisomal membrane protein 2 | 5.20E-05 | -1.135 |
| *RPRD2* | regulation of nuclear pre-mRNA domain containing 2 | 2.00E-05 | -1.140 |
| *ADAM10* | ADAM metallopeptidase domain 10 | 1.74E-04 | -1.150 |
| *XBP1* | X-box binding protein 1 | 2.00E-05 | -1.150 |
| *IGLL3P* | immunoglobulin lambda like polypeptide 3. pseudogene | 5.70E-05 | -1.150 |
| *C10orf2* | twinkle mtDNA helicase | 7.90E-05 | -1.150 |
| *RUNX1* | runt related transcription factor 1 | 2.00E-05 | -1.155 |
| *LCT* | lactase | 2.00E-05 | -1.160 |
| *RHOH* | ras homolog family member H | 6.00E-05 | -1.160 |
| *TMEM19* | transmembrane protein 19 | 1.80E-05 | -1.165 |
| *PVRIG* | PVR related immunoglobulin domain containing | 7.00E-05 | -1.165 |
| *PDCD6IP* | programmed cell death 6 interacting protein | 1.70E-05 | -1.180 |
| *UBL4A* | ubiquitin like 4A | 1.90E-05 | -1.180 |
| *HLTF* | helicase like transcription factor | 3.30E-05 | -1.180 |
| *TIGD5* | tigger transposable element derived 5 | 8.20E-05 | -1.180 |
| *MCAT* | malonyl-CoA-acyl carrier protein transacylase | 1.80E-05 | -1.185 |
| *RRP36* | ribosomal RNA processing 36 | 1.96E-04 | -1.185 |
| *ZMAT3* | zinc finger matrin-type 3 | 2.50E-05 | -1.185 |
| *ANTXR2* | anthrax toxin receptor 2 | 3.10E-05 | -1.190 |
| *UBAP2L* | ubiquitin associated protein 2 like | 3.40E-05 | -1.190 |
| *KLHDC3* | kelch domain containing 3 | 4.70E-05 | -1.190 |
| *DTYMK* | deoxythymidylate kinase | 9.10E-05 | -1.190 |
| *CD1B* | CD1b molecule | 2.60E-05 | -1.195 |
| *GRAP* | GRB2-related adaptor protein | 3.21E-04 | -1.195 |
| *SLC16A3* | solute carrier family 16 member 3 | 1.70E-05 | -1.205 |
| *FAM172A* | family with sequence similarity 172 member A | 2.00E-05 | -1.205 |
| *GEMIN6* | gem nuclear organelle associated protein 6 | 3.50E-05 | -1.205 |
| *CCM2* | CCM2 scaffolding protein | 5.60E-05 | -1.210 |
| *CIRBP* | cold inducible RNA binding protein | 1.40E-05 | -1.215 |
| *RNGTT* | RNA guanylyltransferase and 5'-phosphatase | 1.70E-05 | -1.225 |
| *TMED10* | transmembrane p24 trafficking protein 10 | 2.10E-05 | -1.225 |
| *TMEM203* | transmembrane protein 203 | 2.80E-05 | -1.225 |
| *PAFAH1B1* | platelet activating factor acetylhydrolase 1b regulatory subunit 1 | 1.40E-05 | -1.230 |
| *CPVL* | carboxypeptidase. vitellogenic like | 2.60E-05 | -1.230 |
| *BCAT1* | branched chain amino acid transaminase 1 | 2.90E-05 | -1.230 |
| *ICAM2* | intercellular adhesion molecule 2 | 1.17E-04 | -1.235 |
| *CD93* | CD93 molecule | 1.40E-05 | -1.240 |
| *PAG1* | phosphoprotein membrane anchor with glycosphingolipid microdomains 1 | 2.70E-05 | -1.240 |
| *MIR600HG* | MIR600 host gene | 1.30E-05 | -1.250 |
| *PAGR1* | PAXIP1 associated glutamate rich protein 1 | 2.70E-05 | -1.255 |
| *HDAC1* | histone deacetylase 1 | 2.10E-05 | -1.270 |
| *CDK6* | cyclin dependent kinase 6 | 1.30E-05 | -1.285 |
| *MRPL20* | mitochondrial ribosomal protein L20 | 2.80E-05 | -1.290 |
| *FASN* | fatty acid synthase | 1.30E-05 | -1.300 |
| *D2HGDH* | D-2-hydroxyglutarate dehydrogenase | 1.40E-05 | -1.300 |
| *BCL11A* | B cell CLL/lymphoma 11A | 1.30E-05 | -1.305 |
| *USP24* | ubiquitin specific peptidase 24 | 1.60E-05 | -1.305 |
| *GORASP2* | golgi reassembly stacking protein 2 | 1.40E-05 | -1.310 |
| *JARID2* | jumonji and AT-rich interaction domain containing 2 | 1.00E-05 | -1.325 |
| *PPM1A* | protein phosphatase. Mg2+/Mn2+ dependent 1A | 1.20E-05 | -1.330 |
| *TMEM33* | transmembrane protein 33 | 1.80E-05 | -1.330 |
| *ARID1B* | AT-rich interaction domain 1B | 1.00E-05 | -1.350 |
| *MRPL17* | mitochondrial ribosomal protein L17 | 2.80E-05 | -1.350 |
| *SPC25* | SPC25. NDC80 kinetochore complex component | 2.40E-05 | -1.355 |
| *PHB* | prohibitin | 3.40E-05 | -1.360 |
| *PDK3* | pyruvate dehydrogenase kinase 3 | 1.20E-05 | -1.370 |
| *CCDC167* | coiled-coil domain containing 167 | 4.20E-05 | -1.375 |
| *GPR162* | G protein-coupled receptor 162 | 2.80E-05 | -1.385 |
| *CENPB* | centromere protein B | 7.00E-05 | -1.385 |
| *RAB22A* | RAB22A. member RAS oncogene family | 2.00E-05 | -1.390 |
| *MAEA* | macrophage erythroblast attacher | 1.80E-05 | -1.395 |
| *GEMIN4* | gem nuclear organelle associated protein 4 | 1.00E-05 | -1.420 |
| *ARID2* | AT-rich interaction domain 2 | 6.00E-06 | -1.435 |
| *HNRNPA0* | heterogeneous nuclear ribonucleoprotein A0 | 6.00E-06 | -1.440 |
| *NLGN4X* | neuroligin 4. X-linked | 7.00E-06 | -1.470 |
| *PLCH1* | phospholipase C eta 1 | 2.00E-05 | -1.475 |
| *RAB27A* | RAB27A. member RAS oncogene family | 2.00E-05 | -1.535 |
| *TNFRSF1A* | TNF receptor superfamily member 1A | 2.30E-05 | -1.565 |
| *VPREB1* | V-set pre-B cell surrogate light chain 1 | 5.40E-05 | -1.610 |
| *RIMS3* | regulating synaptic membrane exocytosis 3 | 6.00E-06 | -1.630 |
| *ABCE1* | ATP binding cassette subfamily E member 1 | 2.00E-06 | -1.635 |
| *SLA* | Src like adaptor | 1.70E-05 | -1.640 |
| *UBASH3B* | ubiquitin associated and SH3 domain containing B | 1.10E-05 | -1.650 |
| *BCL11B* | B cell CLL/lymphoma 11B | 4.00E-06 | -1.665 |
| *SLC39A3* | solute carrier family 39 member 3 | 1.70E-05 | -1.775 |

Chipster analysis identified 492 significantly deregulated genes in CCRF-CEM treated cells.
