# Supplementary material for: Cytotoxicity of nimbolide towards multidrug-resistant tumor cells and hypersensitivity via cellular metabolic modulation
Source: Oncotarget. 2018 Nov 6;9(87):35762–79. doi: 10.18632/oncotarget.26299 (PMC6254660; doi:10.18632/oncotarget.26299)
Supplement: Supplementary file 3 [file oncotarget-09-35762-s003.docx]

**Supplementary Table 2.** The most significantly deregulated genes in CEM/ADR5000 cells after nimbolide treatment.

| **Symbol** | **Description** | ***P*-value** | **FC** |
| --- | --- | --- | --- |
| *SNORD46* | small nucleolar RNA. C/D box 46 | 2.00E-06 | 2.540 |
| *MXD4* | MAX dimerization protein 4 | 2.00E-06 | 2.325 |
| *DDIT3* | DNA damage inducible transcript 3 | 2.00E-06 | 2.285 |
| *SNHG7* | small nucleolar RNA host gene 7 | 2.00E-06 | 2.240 |
| *PPP1R15A* | protein phosphatase 1 regulatory subunit 15A | 2.00E-06 | 2.235 |
| *CCNDBP1* | cyclin D1 binding protein 1 | 2.00E-06 | 2.165 |
| *RNU11* | RNA. U11 small nuclear | 2.00E-06 | 2.150 |
| *TUBB3* | tubulin beta 3 class III | 2.00E-06 | 2.145 |
| *SERTAD1* | SERTA domain containing 1 | 2.00E-06 | 2.095 |
| *RASAL3* | RAS protein activator like 3 | 2.00E-06 | 2.095 |
| *YPEL5* | yippee like 5 | 2.00E-06 | 2.080 |
| *MC1R* | melanocortin 1 receptor | 2.00E-06 | 2.075 |
| *SIDT2* | SID1 transmembrane family member 2 | 2.00E-06 | 2.065 |
| *DNAJB2* | DnaJ heat shock protein family (Hsp40) member B2 | 2.00E-06 | 2.060 |
| *SAP25* | Sin3A associated protein 25 | 2.00E-06 | 2.050 |
| *HMOX1* | heme oxygenase 1 | 2.00E-06 | 1.995 |
| *FGFR3* | fibroblast growth factor receptor 3 | 2.00E-06 | 1.945 |
| *PINK1* | PTEN induced putative kinase 1 | 4.00E-06 | 1.920 |
| *MT1F* | metallothionein 1F | 2.00E-06 | 1.885 |
| *ABTB1* | ankyrin repeat and BTB domain containing 1 | 3.00E-06 | 1.880 |
| *SMIM14* | small integral membrane protein 14 | 2.00E-06 | 1.835 |
| *IRF7* | interferon regulatory factor 7 | 2.00E-06 | 1.815 |
| *KLHL24* | kelch like family member 24 | 3.00E-06 | 1.800 |
| *SYNJ1* | synaptojanin 1 | 2.00E-06 | 1.795 |
| *TSC22D3* | TSC22 domain family member 3 | 2.00E-06 | 1.790 |
| *ZDHHC11* | zinc finger DHHC-type containing 11 | 3.00E-06 | 1.760 |
| *TAGLN* | transgelin | 3.00E-06 | 1.745 |
| *ATF5* | activating transcription factor 5 | 3.00E-06 | 1.730 |
| *FAM214A* | family with sequence similarity 214 member A | 3.00E-06 | 1.730 |
| *CDK19* | cyclin dependent kinase 19 | 3.00E-06 | 1.725 |
| *NDRG1* | N-myc downstream regulated 1 | 5.00E-06 | 1.720 |
| *SLC2A3* | solute carrier family 2 member 3 | 2.00E-06 | 1.675 |
| *PMM1* | phosphomannomutase 1 | 2.00E-06 | 1.655 |
| *IDS* | iduronate 2-sulfatase | 3.00E-06 | 1.645 |
| *RNU6ATAC* | RNA. U6atac small nuclear (U12-dependent splicing) | 2.00E-06 | 1.640 |
| *ULK1* | unc-51 like autophagy activating kinase 1 | 2.00E-06 | 1.625 |
| *MAP1LC3B* | microtubule associated protein 1 light chain 3 beta | 3.00E-06 | 1.605 |
| *ANG* | angiogenin | 3.00E-06 | 1.595 |
| *TMEM8B* | transmembrane protein 8B | 2.00E-06 | 1.590 |
| *NXF1* | nuclear RNA export factor 1 | 3.00E-06 | 1.580 |
| *RDH5* | retinol dehydrogenase 5 | 4.00E-06 | 1.580 |
| *CCNG2* | cyclin G2 | 4.00E-06 | 1.560 |
| *ID2* | inhibitor of DNA binding 2 | 3.00E-06 | 1.545 |
| *SCARB2* | scavenger receptor class B member 2 | 3.00E-06 | 1.545 |
| *MGEA5* | meningioma expressed antigen 5 (hyaluronidase) | 4.00E-06 | 1.545 |
| *TEX19* | testis expressed 19 | 5.00E-06 | 1.545 |
| *AAMDC* | adipogenesis associated Mth938 domain containing | 3.00E-06 | 1.520 |
| *GABARAPL1* | GABA type A receptor associated protein like 1 | 3.00E-06 | 1.515 |
| *CSAD* | cysteine sulfinic acid decarboxylase | 3.00E-06 | 1.500 |
| *DNAL4* | dynein axonemal light chain 4 | 4.00E-06 | 1.500 |
| *DHRS2* | dehydrogenase/reductase 2 | 5.00E-06 | 1.500 |
| *SNORD56* | small nucleolar RNA. C/D box 56 | 3.00E-06 | 1.490 |
| *RNU4ATAC* | RNA. U4atac small nuclear (U12-dependent splicing) | 3.00E-06 | 1.485 |
| *BSDC1* | BSD domain containing 1 | 3.00E-06 | 1.480 |
| *CCDC28A* | coiled-coil domain containing 28A | 3.00E-06 | 1.475 |
| *TROAP* | trophinin associated protein | 3.00E-06 | 1.470 |
| *CLDN15* | claudin 15 | 4.00E-06 | 1.470 |
| *BSN* | bassoon presynaptic cytomatrix protein | 3.00E-06 | 1.465 |
| *HIST1H2AC* | histone cluster 1 H2A family member c | 3.00E-06 | 1.460 |
| *SNORD12C* | small nucleolar RNA. C/D box 12C | 5.00E-06 | 1.460 |
| *FLJ20021* | uncharacterized LOC90024 | 4.00E-06 | 1.455 |
| *CTDSP2* | CTD small phosphatase 2 | 3.00E-06 | 1.445 |
| *ZSWIM8* | zinc finger SWIM-type containing 8 | 3.00E-06 | 1.435 |
| *UBALD2* | UBA like domain containing 2 | 1.00E-05 | 1.435 |
| *SLC44A1* | solute carrier family 44 member 1 | 3.00E-06 | 1.430 |
| *CSNK2A2* | casein kinase 2 alpha 2 | 3.00E-06 | 1.430 |
| *EIF1* | eukaryotic translation initiation factor 1 | 6.00E-06 | 1.425 |
| *TXNIP* | thioredoxin interacting protein | 6.00E-06 | 1.425 |
| *RNF103* | ring finger protein 103 | 6.00E-06 | 1.425 |
| *TPRG1L* | tumor protein p63 regulated 1 like | 3.00E-06 | 1.410 |
| *NIPAL3* | NIPA like domain containing 3 | 6.00E-06 | 1.410 |
| *CHIC2* | cysteine rich hydrophobic domain 2 | 8.00E-06 | 1.410 |
| *MPPE1* | metallophosphoesterase 1 | 3.00E-06 | 1.405 |
| *COL7A1* | collagen type VII alpha 1 chain | 3.00E-06 | 1.400 |
| *TMBIM1* | transmembrane BAX inhibitor motif containing 1 | 3.00E-06 | 1.400 |
| *MT1H* | metallothionein 1H | 4.00E-06 | 1.400 |
| *GPR137* | G protein-coupled receptor 137 | 3.00E-06 | 1.395 |
| *PCNX* | pecanex homolog 1 | 3.00E-06 | 1.390 |
| *CREBRF* | CREB3 regulatory factor | 4.00E-06 | 1.385 |
| *GLIPR2* | GLI pathogenesis related 2 | 4.00E-06 | 1.375 |
| *BNIPL* | BCL2 interacting protein like | 3.00E-06 | 1.370 |
| *HELZ2* | helicase with zinc finger 2 | 6.00E-06 | 1.360 |
| *KIAA1683* | IQ motif containing N | 3.00E-06 | 1.355 |
| *CALM1* | calmodulin 1 | 5.00E-06 | 1.355 |
| *TUBB2A* | tubulin beta 2A class IIa | 7.00E-06 | 1.355 |
| *KIZ* | kizuna centrosomal protein | 2.30E-05 | 1.355 |
| *TMOD1* | tropomodulin 1 | 4.00E-06 | 1.345 |
| *AGTPBP1* | ATP/GTP binding protein 1 | 6.00E-06 | 1.340 |
| *MXD1* | MAX dimerization protein 1 | 1.10E-05 | 1.340 |
| *NEU1* | neuraminidase 1 | 3.00E-06 | 1.335 |
| *LY96* | lymphocyte antigen 96 | 5.00E-06 | 1.335 |
| *NDE1* | nudE neurodevelopment protein 1 | 5.00E-06 | 1.335 |
| *OSER1* | oxidative stress responsive serine rich 1 | 9.00E-06 | 1.335 |
| *STAT2* | signal transducer and activator of transcription 2 | 5.00E-06 | 1.325 |
| *TM2D1* | TM2 domain containing 1 | 3.00E-06 | 1.325 |
| *NCOA7* | nuclear receptor coactivator 7 | 7.00E-06 | 1.325 |
| *LRP10* | LDL receptor related protein 10 | 4.00E-06 | 1.320 |
| *TRIM68* | tripartite motif containing 68 | 5.00E-06 | 1.310 |
| *SERPINI1* | serpin family I member 1 | 5.00E-06 | 1.310 |
| *RAB5B* | RAB5B. member RAS oncogene family | 4.00E-06 | 1.305 |
| *SNORD12* | small nucleolar RNA. C/D box 12 | 8.00E-06 | 1.305 |
| *SWI5* | SWI5 homologous recombination repair protein | 4.00E-06 | 1.300 |
| *SCPEP1* | serine carboxypeptidase 1 | 5.00E-06 | 1.300 |
| *SYF2* | SYF2 pre-mRNA splicing factor | 6.00E-06 | 1.300 |
| *FTH1* | ferritin heavy chain 1 | 4.00E-06 | 1.290 |
| *MEF2D* | myocyte enhancer factor 2D | 6.00E-06 | 1.290 |
| *CAPN5* | calpain 5 | 3.80E-05 | 1.285 |
| *ANKRD36* | ankyrin repeat domain 36 | 1.50E-05 | 1.280 |
| *NEK2* | NIMA related kinase 2 | 5.00E-06 | 1.275 |
| *CLIP3* | CAP-Gly domain containing linker protein 3 | 6.00E-06 | 1.275 |
| *RILPL1* | Rab interacting lysosomal protein like 1 | 2.40E-05 | 1.275 |
| *ASNS* | asparagine synthetase (glutamine-hydrolyzing) | 4.00E-06 | 1.260 |
| *AKR1C3* | aldo-keto reductase family 1 member C3 | 8.00E-06 | 1.255 |
| *WDR26* | WD repeat domain 26 | 1.00E-05 | 1.255 |
| *FBXL18* | F-box and leucine rich repeat protein 18 | 5.00E-06 | 1.235 |
| *NPC2* | NPC intracellular cholesterol transporter 2 | 4.00E-06 | 1.230 |
| *NBPF10* | NBPF member 20 | 4.00E-06 | 1.230 |
| *PIAS4* | protein inhibitor of activated STAT 4 | 4.00E-06 | 1.225 |
| *C11orf54* | chromosome 11 open reading frame 54 | 4.00E-06 | 1.225 |
| *NPC1* | NPC intracellular cholesterol transporter 1 | 9.00E-06 | 1.225 |
| *YTHDC1* | YTH domain containing 1 | 1.00E-05 | 1.225 |
| *SERINC1* | serine incorporator 1 | 6.00E-06 | 1.220 |
| *GFOD1* | glucose-fructose oxidoreductase domain containing 1 | 7.00E-06 | 1.220 |
| *HSPA1B* | heat shock protein family A (Hsp70) member 1A | 5.00E-06 | 1.215 |
| *SAT1* | spermidine/spermine N1-acetyltransferase 1 | 7.00E-06 | 1.215 |
| *BRPF3* | bromodomain and PHD finger containing 3 | 9.00E-06 | 1.210 |
| *VAMP1* | vesicle associated membrane protein 1 | 9.00E-06 | 1.210 |
| *WDR20* | WD repeat domain 20 | 1.20E-05 | 1.210 |
| *SNX30* | sorting nexin family member 30 | 1.60E-05 | 1.210 |
| *PQLC3* | PQ loop repeat containing 3 | 6.00E-06 | 1.210 |
| *AKAP8L* | A-kinase anchoring protein 8 like | 3.80E-05 | 1.205 |
| *RSRP1* | arginine and serine rich protein 1 | 4.60E-05 | 1.200 |
| *TSPAN9* | tetraspanin 9 | 4.00E-06 | 1.195 |
| *SAP30L* | SAP30 like | 8.00E-06 | 1.195 |
| *BCL6* | B cell CLL/lymphoma 6 | 4.60E-05 | 1.195 |
| *WDR19* | WD repeat domain 19 | 5.00E-06 | 1.190 |
| *POLR3GL* | RNA polymerase III subunit G like | 5.00E-06 | 1.185 |
| *DEF8* | differentially expressed in FDCP 8 homolog | 5.00E-06 | 1.185 |
| *GNS* | glucosamine (N-acetyl)-6-sulfatase | 5.00E-06 | 1.185 |
| *PCMTD1* | protein-L-isoaspartate (D-aspartate) O-methyltransferase domain containing 1 | 1.00E-05 | 1.185 |
| *TTLL3* | tubulin tyrosine ligase like 3 | 5.00E-06 | 1.175 |
| *CDCA8* | cell division cycle associated 8 | 8.00E-06 | 1.175 |
| *TOM1* | target of myb1 membrane trafficking protein | 1.10E-05 | 1.175 |
| *FIG4* | FIG4 phosphoinositide 5-phosphatase | 5.00E-06 | 1.170 |
| *SLC25A20* | solute carrier family 25 member 20 | 8.00E-06 | 1.170 |
| *IFRD1* | interferon related developmental regulator 1 | 1.10E-05 | 1.170 |
| *BBS4* | Bardet-Biedl syndrome 4 | 5.00E-06 | 1.165 |
| *NOA1* | nitric oxide associated 1 | 6.00E-06 | 1.160 |
| *SNORA28* | small nucleolar RNA. H/ACA box 28 | 2.00E-05 | 1.155 |
| *RETSAT* | retinol saturase | 2.60E-05 | 1.155 |
| *CENPA* | centromere protein A | 5.00E-06 | 1.150 |
| *FTH1P3* | ferritin heavy chain 1 pseudogene 3 | 6.00E-06 | 1.150 |
| *IRF9* | interferon regulatory factor 9 | 5.00E-06 | 1.145 |
| *ZSWIM6* | zinc finger SWIM-type containing 6 | 3.80E-05 | 1.145 |
| *NCOA1* | nuclear receptor coactivator 1 | 5.00E-06 | 1.140 |
| *ANKRD36B* | ankyrin repeat domain 36B | 1.00E-05 | 1.140 |
| *AP4B1* | adaptor related protein complex 4 beta 1 subunit | 5.00E-06 | 1.135 |
| *HIST1H4A* | histone cluster 1 H4 family member a | 6.00E-06 | 1.135 |
| *RAB2B* | RAB2B. member RAS oncogene family | 6.00E-06 | 1.130 |
| *RRAGC* | Ras related GTP binding C | 6.00E-06 | 1.130 |
| *IL11RA* | interleukin 11 receptor subunit alpha | 1.20E-05 | 1.125 |
| *SPSB3* | splA/ryanodine receptor domain and SOCS box containing 3 | 6.00E-06 | 1.120 |
| *SNHG6* | small nucleolar RNA host gene 6 | 7.00E-06 | 1.120 |
| *DCTN3* | dynactin subunit 3 | 8.00E-06 | 1.120 |
| *EXT2* | exostosin glycosyltransferase 2 | 1.30E-05 | 1.120 |
| *RCOR2* | REST corepressor 2 | 1.80E-05 | 1.120 |
| *PNISR* | PNN interacting serine and arginine rich protein | 2.40E-05 | 1.120 |
| *TUBB7P* | tubulin beta 7 pseudogene | 6.00E-06 | 1.115 |
| *LRRFIP2* | LRR binding FLII interacting protein 2 | 1.50E-05 | 1.115 |
| *YIPF1* | Yip1 domain family member 1 | 1.70E-05 | 1.115 |
| *FAM149B1* | family with sequence similarity 149 member B1 | 6.10E-05 | 1.115 |
| *LMO4* | LIM domain only 4 | 6.00E-06 | 1.110 |
| *DBNDD1* | dysbindin domain containing 1 | 8.00E-06 | 1.110 |
| *RGS2* | regulator of G protein signaling 2 | 9.00E-06 | 1.110 |
| *KIAA0355* | KIAA0355 | 1.00E-05 | 1.110 |
| *ZBTB43* | zinc finger and BTB domain containing 43 | 1.20E-05 | 1.110 |
| *NCOA3* | nuclear receptor coactivator 3 | 6.00E-06 | 1.105 |
| *RRAS* | RAS related | 6.00E-06 | 1.105 |
| *OPTN* | optineurin | 1.15E-04 | 1.105 |
| *GSDMB* | gasdermin B | 8.20E-05 | 1.100 |
| *MLLT4* | afadin. adherens junction formation factor | 6.00E-06 | 1.100 |
| *SEMA4F* | ssemaphorin 4F | 1.50E-05 | 1.100 |
| *ARHGAP33* | Rho GTPase activating protein 33 | 5.50E-05 | 1.095 |
| *TESK2* | testis-specific kinase 2 | 8.00E-06 | 1.090 |
| *BTG1* | BTG anti-proliferation factor 1 | 1.10E-05 | 1.090 |
| *IFIT2* | interferon induced protein with tetratricopeptide repeats 2 | 4.70E-05 | 1.090 |
| *EFR3B* | EFR3 homolog B | 8.00E-06 | 1.085 |
| *PIGH* | phosphatidylinositol glycan anchor biosynthesis class H | 2.60E-05 | 1.085 |
| *LINC01137* | long intergenic non-protein coding RNA 1137 | 2.80E-05 | 1.085 |
| *ALB* | albumin | 6.00E-06 | 1.085 |
| *CDKN2D* | cyclin dependent kinase inhibitor 2D | 6.00E-06 | 1.085 |
| *RBM5* | RNA binding motif protein 5 | 7.00E-06 | 1.080 |
| *TMEM41B* | transmembrane protein 41B | 8.00E-06 | 1.080 |
| *IFI6* | interferon alpha inducible protein 6 | 1.20E-05 | 1.080 |
| *UBE2H* | ubiquitin conjugating enzyme E2 H | 1.20E-05 | 1.080 |
| *PIK3IP1* | phosphoinositide-3-kinase interacting protein 1 | 5.10E-05 | 1.080 |
| *LMBRD1* | LMBR1 domain containing 1 | 1.00E-05 | 1.075 |
| *AMH* | anti-Mullerian hormone | 1.00E-05 | 1.075 |
| *GSTA4* | glutathione S-transferase alpha 4 | 3.70E-05 | 1.075 |
| *FAM53C* | family with sequence similarity 53 member C | 6.00E-06 | 1.070 |
| *MTMR14* | myotubularin related protein 14 | 8.00E-06 | 1.070 |
| *ATP6V0A1* | ATPase H+ transporting V0 subunit a1 | 9.00E-06 | 1.070 |
| *HMG20B* | high mobility group 20B | 1.00E-05 | 1.070 |
| *ATP6V1D* | ATPase H+ transporting V1 subunit D | 1.40E-05 | 1.070 |
| *BRI3* | brain protein I3 | 6.00E-06 | 1.065 |
| *ZC2HC1A* | zinc finger C2HC-type containing 1A | 8.00E-06 | 1.060 |
| *MAGED2* | MAGE family member D2 | 7.50E-05 | 1.060 |
| *NRDE2* | NRDE-2. necessary for RNA interference. domain containing | 7.00E-06 | 1.050 |
| *DSTYK* | dual serine/threonine and tyrosine protein kinase | 7.00E-06 | 1.050 |
| *TMEM91* | transmembrane protein 91 | 8.00E-06 | 1.050 |
| *LHFP* | LHFPL tetraspan subfamily member 6 | 9.00E-06 | 1.050 |
| *RBMS1* | RNA binding motif single stranded interacting protein 1 | 8.00E-06 | 1.045 |
| *UPF3A* | UPF3A. regulator of nonsense mediated mRNA decay | 9.00E-06 | 1.045 |
| *HMMR* | hyaluronan mediated motility receptor | 1.40E-05 | 1.045 |
| *LCOR* | ligand dependent nuclear receptor corepressor | 1.90E-05 | 1.045 |
| *SLC35A1* | solute carrier family 35 member A1 | 9.80E-05 | 1.045 |
| *HOXB7* | homeobox B7 | 8.00E-06 | 1.040 |
| *CDKN2C* | cyclin dependent kinase inhibitor 2C | 8.00E-06 | 1.040 |
| *TRAK2* | trafficking kinesin protein 2 | 2.70E-05 | 1.040 |
| *CCPG1* | cell cycle progression 1 | 1.00E-05 | 1.035 |
| *CGGBP1* | CGG triplet repeat binding protein 1 | 1.20E-05 | 1.030 |
| *CBLB* | Cbl proto-oncogene B | 1.80E-05 | 1.030 |
| *SQSTM1* | sequestosome 1 | 8.00E-06 | 1.025 |
| *ATP6V0D1* | ATPase H+ transporting V0 subunit d1 | 1.00E-05 | 1.025 |
| *RPS2* | ribosomal protein S2 | 2.70E-05 | 1.025 |
| *NDFIP2* | Nedd4 family interacting protein 2 | 2.80E-05 | 1.025 |
| *MT1G* | metallothionein 1G | 9.00E-06 | 1.020 |
| *KIFAP3* | kinesin associated protein 3 | 9.00E-06 | 1.015 |
| *RTN1* | reticulon 1 | 9.00E-06 | 1.015 |
| *ALOX5AP* | arachidonate 5-lipoxygenase activating protein | 1.00E-05 | 1.015 |
| *ABHD12* | abhydrolase domain containing 12 | 1.10E-05 | 1.015 |
| *RAPGEF1* | Rap guanine nucleotide exchange factor 1 | 1.90E-05 | 1.015 |
| *CCDC93* | coiled-coil domain containing 93 | 8.00E-06 | 1.010 |
| *UCN* | urocortin | 8.00E-06 | 1.010 |
| *CHMP5* | charged multivesicular body protein 5 | 1.40E-05 | 1.010 |
| *FAM172A* | family with sequence similarity 172 member A | 1.40E-05 | 1.010 |
| *AHSA2* | activator of HSP90 ATPase homolog 2. pseudogene | 1.80E-05 | 1.010 |
| *ANKRD12* | ankyrin repeat domain 12 | 2.30E-05 | 1.010 |
| *RPPH1* | ribonuclease P RNA component H1 | 7.40E-05 | 1.010 |
| *CCDC130* | coiled-coil domain containing 130 | 1.00E-05 | 1.005 |
| *SETX* | senataxin | 1.10E-05 | 1.005 |
| *KLF6* | Kruppel like factor 6 | 9.00E-06 | 1.005 |
| *ZNF277* | zinc finger protein 277 | 1.30E-05 | 1.005 |
| *ZNF451* | zinc finger protein 451 | 2.40E-05 | 1.005 |
| *CENPL* | centromere protein L | 5.26E-04 | 1.005 |
| *ERGIC3* | ERGIC and golgi 3 | 2.20E-05 | 1.000 |
| *LPAR2* | lysophosphatidic acid receptor 2 | 2.20E-05 | 1.000 |
| *BRD8* | bromodomain containing 8 | 2.90E-05 | 1.000 |
| *ZSWIM4* | zinc finger SWIM-type containing 4 | 3.70E-05 | 1.000 |
| *DNAJB4* | DnaJ heat shock protein family (Hsp40) member B4 | 1.76E-04 | 1.000 |
| *DCAF13* | DDB1 and CUL4 associated factor 13 | 3.30E-05 | -1.000 |
| *PDCD2L* | programmed cell death 2 like | 2.20E-05 | -1.000 |
| *TATDN2* | TatD DNase domain containing 2 | 1.00E-05 | -1.005 |
| *CALML4* | calmodulin like 4 | 2.50E-05 | -1.005 |
| *GYPC* | glycophorin C (Gerbich blood group) | 2.60E-05 | -1.005 |
| *ICAM2* | intercellular adhesion molecule 2 | 1.02E-04 | -1.005 |
| *POTEKP* | POTE ankyrin domain family member K. pseudogene | 4.60E-05 | -1.010 |
| *ACACA* | acetyl-CoA carboxylase alpha | 9.00E-06 | -1.010 |
| *DDX28* | DEAD-box helicase 28 | 3.94E-04 | -1.010 |
| *LARP1* | La ribonucleoprotein domain family member 1 | 9.00E-06 | -1.010 |
| *SLC25A15* | solute carrier family 25 member 15 | 9.00E-06 | -1.010 |
| *MCM6* | minichromosome maintenance complex component 6 | 1.10E-05 | -1.010 |
| *UNC93B1* | unc-93 homolog B1. TLR signaling regulator | 8.00E-06 | -1.015 |
| *ELMO1* | engulfment and cell motility 1 | 1.10E-05 | -1.015 |
| *CHAF1B* | chromatin assembly factor 1 subunit B | 3.40E-05 | -1.015 |
| *H2AFY* | H2A histone family member Y | 5.90E-05 | -1.015 |
| *CD47* | CD47 molecule | 8.00E-06 | -1.020 |
| *SOCS2* | suppressor of cytokine signaling 2 | 8.00E-06 | -1.020 |
| *HSPA9* | heat shock protein family A (Hsp70) member 9 | 1.10E-05 | -1.020 |
| *SLC29A4* | solute carrier family 29 member 4 | 1.20E-05 | -1.020 |
| *TTF2* | transcription termination factor 2 | 8.00E-06 | -1.020 |
| *ENOPH1* | enolase-phosphatase 1 | 8.00E-06 | -1.020 |
| *RHOU* | ras homolog family member U | 9.00E-06 | -1.020 |
| *ATP5G3* | ATP synthase membrane subunit c locus 3 | 2.50E-05 | -1.020 |
| *TMEM109* | transmembrane protein 109 | 2.60E-05 | -1.020 |
| *PIK3AP1* | phosphoinositide-3-kinase adaptor protein 1 | 2.90E-05 | -1.020 |
| *SREBF1* | sterol regulatory element binding transcription factor 1 | 8.00E-06 | -1.025 |
| *ACTN1* | actinin alpha 1 | 9.00E-06 | -1.025 |
| *DHX15* | DEAH-box helicase 15 | 1.00E-05 | -1.025 |
| *KBTBD11* | kelch repeat and BTB domain containing 11 | 1.40E-05 | -1.025 |
| *GTF3C6* | general transcription factor IIIC subunit 6 | 2.50E-05 | -1.025 |
| *AEBP1* | AE binding protein 1 | 1.28E-04 | -1.025 |
| *PSMB10* | proteasome subunit beta 10 | 8.00E-06 | -1.025 |
| *LOC644936* | actin beta pseudogene | 8.00E-06 | -1.025 |
| *PMM2* | phosphomannomutase 2 | 9.00E-06 | -1.030 |
| *CCT2* | chaperonin containing TCP1 subunit 2 | 9.00E-06 | -1.030 |
| *DDX47* | DEAD-box helicase 47 | 1.00E-05 | -1.030 |
| *PSPC1* | paraspeckle component 1 | 1.90E-05 | -1.030 |
| *KPNA3* | karyopherin subunit alpha 3 | 2.30E-05 | -1.030 |
| *RRP1B* | ribosomal RNA processing 1B | 3.90E-05 | -1.030 |
| *NDUFAF3* | NADH:ubiquinone oxidoreductase complex assembly factor 3 | 8.00E-06 | -1.030 |
| *TRAT1* | T cell receptor associated transmembrane adaptor 1 | 1.00E-05 | -1.030 |
| *PSME2* | proteasome activator subunit 2 | 1.10E-05 | -1.030 |
| *ANP32C* | acidic nuclear phosphoprotein 32 family member C | 1.10E-05 | -1.030 |
| *CORO1A* | coronin 1A | 1.30E-05 | -1.030 |
| *GRPEL1* | GrpE like 1. mitochondrial | 2.00E-05 | -1.030 |
| *VTRNA1-3* | vault RNA 1-3 | 2.53E-04 | -1.030 |
| *SAMSN1* | SAM domain. SH3 domain and nuclear localization signals 1 | 5.35E-04 | -1.030 |
| *NAT10* | N-acetyltransferase 10 | 8.00E-06 | -1.035 |
| *CAD* | carbamoyl-phosphate synthetase 2. aspartate transcarbamylase. and dihydroorotase | 9.00E-06 | -1.035 |
| *NUP210* | nucleoporin 210 | 2.00E-05 | -1.035 |
| *MSI2* | musashi RNA binding protein 2 | 3.20E-05 | -1.035 |
| *MRPL1* | mitochondrial ribosomal protein L1 | 7.00E-06 | -1.040 |
| *LCT* | lactase | 8.00E-06 | -1.040 |
| *MLLT6* | MLLT6. PHD finger containing | 2.70E-05 | -1.040 |
| *CD3D* | CD3d molecule | 1.00E-05 | -1.045 |
| *PLEKHG4* | pleckstrin homology and RhoGEF domain containing G4 | 4.30E-05 | -1.045 |
| *LYPLA1* | lysophospholipase I | 1.10E-05 | -1.045 |
| *NFE2* | nuclear factor. erythroid 2 | 7.00E-06 | -1.050 |
| *STEAP3* | STEAP3 metalloreductase | 7.00E-06 | -1.050 |
| *CYC1* | cytochrome c1 | 7.00E-06 | -1.050 |
| *TMEM223* | transmembrane protein 223 | 8.00E-06 | -1.050 |
| *F12* | coagulation factor XII | 1.10E-05 | -1.050 |
| *CRTAP* | cartilage associated protein | 4.00E-05 | -1.050 |
| *NIP7* | NIP7. nucleolar pre-rRNA processing protein | 1.00E-05 | -1.055 |
| *MRPL3* | mitochondrial ribosomal protein L3 | 6.00E-06 | -1.055 |
| *NDUFAF6* | NADH:ubiquinone oxidoreductase complex assembly factor 6 | 1.20E-05 | -1.055 |
| *FAM60A* | SIN3-HDAC complex associated factor | 2.40E-05 | -1.055 |
| *LARS* | leucyl-tRNA synthetase | 7.00E-06 | -1.060 |
| *PFN1* | profilin 1 | 7.00E-06 | -1.060 |
| *PM20D2* | peptidase M20 domain containing 2 | 9.00E-06 | -1.060 |
| *SNORD36A* | small nucleolar RNA. C/D box 36A | 2.00E-05 | -1.060 |
| *PPP2CA* | protein phosphatase 2 catalytic subunit alpha | 9.00E-06 | -1.065 |
| *RFX5* | regulatory factor X5 | 9.00E-06 | -1.065 |
| *SIX4* | SIX homeobox 4 | 1.00E-05 | -1.065 |
| *RNMTL1* | mitochondrial rRNA methyltransferase 3 | 1.00E-05 | -1.065 |
| *SORD* | sorbitol dehydrogenase | 1.30E-05 | -1.065 |
| *GPX7* | glutathione peroxidase 7 | 1.00E-05 | -1.065 |
| *DLAT* | dihydrolipoamide S-acetyltransferase | 1.00E-05 | -1.065 |
| *BID* | BH3 interacting domain death agonist | 1.10E-05 | -1.065 |
| *IMPDH1* | inosine monophosphate dehydrogenase 1 | 1.10E-05 | -1.065 |
| *ESYT1* | extended synaptotagmin 1 | 7.00E-06 | -1.070 |
| *CTSG* | cathepsin G | 9.00E-06 | -1.070 |
| *CRACR2B* | calcium release activated channel regulator 2B | 1.10E-05 | -1.070 |
| *PRKDC* | protein kinase. DNA-activated. catalytic polypeptide | 1.10E-05 | -1.070 |
| *FAM60CP* | family with sequence similarity 60 member C. pseudogene | 1.30E-05 | -1.070 |
| *MCM10* | minichromosome maintenance 10 replication initiation factor | 3.20E-05 | -1.075 |
| *NOS3* | nitric oxide synthase 3 | 6.00E-06 | -1.080 |
| *RAD23B* | RAD23 homolog B. nucleotide excision repair protein | 1.50E-05 | -1.080 |
| *SMYD2* | SET and MYND domain containing 2 | 2.00E-05 | -1.080 |
| *POU4F1* | POU class 4 homeobox 1 | 2.60E-05 | -1.080 |
| *SLC25A13* | solute carrier family 25 member 13 | 6.60E-05 | -1.080 |
| *PP7080* | uncharacterized LOC25845 | 5.30E-04 | -1.080 |
| *AMD1* | adenosylmethionine decarboxylase 1 | 9.00E-06 | -1.085 |
| *SNU13* | small nuclear ribonucleoprotein 13 | 5.10E-05 | -1.085 |
| *NME2* | NME/NM23 nucleoside diphosphate kinase 2 | 4.70E-05 | -1.085 |
| *CDK6* | cyclin dependent kinase 6 | 6.00E-06 | -1.090 |
| *KRT40* | keratin 40 | 7.00E-06 | -1.090 |
| *NASP* | nuclear autoantigenic sperm protein | 1.00E-05 | -1.090 |
| *SRSF3* | serine and arginine rich splicing factor 3 | 5.40E-05 | -1.090 |
| *UCP2* | uncoupling protein 2 | 6.00E-06 | -1.095 |
| *MED16* | mediator complex subunit 16 | 7.00E-06 | -1.095 |
| *SLC38A10* | solute carrier family 38 member 10 | 7.00E-06 | -1.095 |
| *STUB1* | STIP1 homology and U-box containing protein 1 | 1.00E-05 | -1.095 |
| *MCM3AP-AS1* | MCM3AP antisense RNA 1 | 1.40E-05 | -1.095 |
| *CXCR4* | C-X-C motif chemokine receptor 4 | 7.50E-05 | -1.095 |
| *NOP14* | NOP14 nucleolar protein | 8.00E-06 | -1.100 |
| *NHP2* | NHP2 ribonucleoprotein | 1.60E-05 | -1.100 |
| *FERMT3* | fermitin family member 3 | 7.00E-06 | -1.100 |
| *NCS1* | neuronal calcium sensor 1 | 2.40E-05 | -1.100 |
| *PEX10* | peroxisomal biogenesis factor 10 | 2.12E-04 | -1.105 |
| *HRSP12* | reactive intermediate imine deaminase A homolog | 2.10E-05 | -1.110 |
| *SLC39A10* | solute carrier family 39 member 10 | 8.00E-06 | -1.110 |
| *CD44* | CD44 molecule (Indian blood group) | 2.50E-05 | -1.110 |
| *VAV3* | vav guanine nucleotide exchange factor 3 | 1.77E-04 | -1.110 |
| *FAS* | Fas cell surface death receptor | 1.10E-05 | -1.115 |
| *RAB33A* | RAB33A. member RAS oncogene family | 2.70E-05 | -1.115 |
| *ATP1A1* | ATPase Na+/K+ transporting subunit alpha 1 | 3.20E-05 | -1.115 |
| *AFF3* | AF4/FMR2 family member 3 | 8.00E-06 | -1.115 |
| *EFTUD2* | elongation factor Tu GTP binding domain containing 2 | 8.00E-06 | -1.115 |
| *RCAN2* | regulator of calcineurin 2 | 1.00E-05 | -1.120 |
| *TTC38* | tetratricopeptide repeat domain 38 | 1.40E-05 | -1.120 |
| *RBBP9* | RB binding protein 9. serine hydrolase | 1.60E-05 | -1.120 |
| *GNAQ* | G protein subunit alpha q | 2.57E-04 | -1.120 |
| *RPF2* | ribosome production factor 2 homolog | 1.20E-05 | -1.125 |
| *CACNA1H* | calcium voltage-gated channel subunit alpha1 H | 2.60E-05 | -1.125 |
| *SIGMAR1* | sigma non-opioid intracellular receptor 1 | 9.30E-05 | -1.125 |
| *TRIP13* | thyroid hormone receptor interactor 13 | 1.40E-05 | -1.130 |
| *ANKRD37* | ankyrin repeat domain 37 | 1.25E-03 | -1.130 |
| *PDCL3* | phosducin like 3 | 9.00E-06 | -1.130 |
| *WWC1* | WW and C2 domain containing 1 | 2.80E-05 | -1.130 |
| *FBLN2* | fibulin 2 | 7.00E-06 | -1.135 |
| *DDX46* | DEAD-box helicase 46 | 1.10E-05 | -1.135 |
| *IMP4* | IMP4. U3 small nucleolar ribonucleoprotein | 1.10E-05 | -1.135 |
| *RPS26* | ribosomal protein S26 | 4.60E-05 | -1.135 |
| *NXT1* | nuclear transport factor 2 like export factor 1 | 5.00E-06 | -1.140 |
| *DUS3L* | dihydrouridine synthase 3 like | 6.00E-06 | -1.140 |
| *ACLY* | ATP citrate lyase | 2.00E-05 | -1.140 |
| *PRR13* | proline rich 13 | 5.00E-06 | -1.140 |
| *PPIL1* | peptidylprolyl isomerase like 1 | 1.10E-05 | -1.140 |
| *PTPRCAP* | protein tyrosine phosphatase. receptor type C associated protein | 1.10E-05 | -1.145 |
| *DDX31* | DEAD-box helicase 31 | 2.70E-05 | -1.145 |
| *MIR17HG* | miR-17-92a-1 cluster host gene | 8.00E-06 | -1.150 |
| *CCDC58* | coiled-coil domain containing 58 | 1.50E-05 | -1.155 |
| *DENND1A* | DENN domain containing 1A | 9.00E-06 | -1.165 |
| *MCM4* | minichromosome maintenance complex component 4 | 6.00E-06 | -1.165 |
| *MRPL17* | mitochondrial ribosomal protein L17 | 1.00E-05 | -1.170 |
| *EIF5B* | eukaryotic translation initiation factor 5B | 1.20E-05 | -1.170 |
| *PTRH2* | peptidyl-tRNA hydrolase 2 | 1.80E-05 | -1.175 |
| *ZHX2* | zinc fingers and homeoboxes 2 | 7.00E-06 | -1.175 |
| *FBXO4* | F-box protein 4 | 3.90E-05 | -1.175 |
| *PHF19* | PHD finger protein 19 | 1.10E-05 | -1.180 |
| *NT5DC2* | 5'-nucleotidase domain containing 2 | 1.50E-05 | -1.180 |
| *ODC1* | ornithine decarboxylase 1 | 8.00E-06 | -1.180 |
| *PPIA* | peptidylprolyl isomerase A | 1.20E-05 | -1.180 |
| *KCNQ2* | potassium voltage-gated channel subfamily Q member 2 | 1.80E-05 | -1.180 |
| *CFD* | complement factor D | 5.00E-06 | -1.185 |
| *CCND1* | cyclin D1 | 1.90E-05 | -1.185 |
| *CISD1* | CDGSH iron sulfur domain 1 | 2.10E-05 | -1.185 |
| *TFDP2* | transcription factor Dp-2 | 4.80E-05 | -1.185 |
| *ENO1* | enolase 1 | 1.00E-05 | -1.190 |
| *PPP1R35* | protein phosphatase 1 regulatory subunit 35 | 8.00E-05 | -1.190 |
| *ANP32AP1* | acidic nuclear phosphoprotein 32 family member A pseudogene 1 | 7.00E-06 | -1.190 |
| *EIF4A1* | eukaryotic translation initiation factor 4A1 | 8.00E-06 | -1.190 |
| *WDR90* | WD repeat domain 90 | 4.90E-05 | -1.190 |
| *ITM2A* | integral membrane protein 2A | 5.00E-06 | -1.195 |
| *PAQR4* | progestin and adipoQ receptor family member 4 | 1.10E-05 | -1.195 |
| *HMGB1* | high mobility group box 1 | 1.10E-05 | -1.195 |
| *ALYREF* | Aly/REF export factor | 7.80E-05 | -1.195 |
| *GIMAP6* | GTPase. IMAP family member 6 | 5.00E-06 | -1.195 |
| *P2RY8* | P2Y receptor family member 8 | 7.10E-05 | -1.195 |
| *MAPKAPK3* | mitogen-activated protein kinase-activated protein kinase 3 | 5.00E-06 | -1.200 |
| *PTPRF* | protein tyrosine phosphatase. receptor type F | 5.00E-06 | -1.200 |
| *PSMB8* | proteasome subunit beta 8 | 5.00E-06 | -1.200 |
| *PGAM5* | PGAM family member 5. mitochondrial serine/threonine protein phosphatase | 8.00E-06 | -1.200 |
| *PRDX2* | peroxiredoxin 2 | 1.10E-05 | -1.200 |
| *VASH2* | vasohibin 2 | 6.00E-06 | -1.205 |
| *RBM12* | RNA binding motif protein 12 | 2.00E-05 | -1.205 |
| *ASIC1* | acid sensing ion channel subunit 1 | 1.10E-05 | -1.210 |
| *PRKAR1B* | protein kinase cAMP-dependent type I regulatory subunit beta | 6.40E-05 | -1.210 |
| *RPL36A* | ribosomal protein L36a | 7.00E-06 | -1.215 |
| *ATAD3A* | ATPase family. AAA domain containing 3A | 5.00E-06 | -1.215 |
| *PHF23* | PHD finger protein 23 | 5.00E-06 | -1.215 |
| *CD3G* | CD3g molecule | 5.00E-06 | -1.220 |
| *UCHL5* | ubiquitin C-terminal hydrolase L5 | 5.00E-06 | -1.220 |
| *PNPO* | pyridoxamine 5'-phosphate oxidase | 1.20E-05 | -1.220 |
| *HNRNPU* | heterogeneous nuclear ribonucleoprotein U | 4.00E-06 | -1.225 |
| *CMSS1* | cms1 ribosomal small subunit homolog (yeast) | 1.00E-05 | -1.225 |
| *RCC2* | regulator of chromosome condensation 2 | 4.00E-06 | -1.230 |
| *DDX10* | DEAD-box helicase 10 | 4.00E-06 | -1.230 |
| *BCLAF1* | BCL2 associated transcription factor 1 | 1.30E-05 | -1.230 |
| *PGAM1P8* | phosphoglycerate mutase 1 pseudogene 8 | 4.00E-06 | -1.235 |
| *KAT2A* | lysine acetyltransferase 2A | 7.00E-06 | -1.235 |
| *PPAT* | phosphoribosyl pyrophosphate amidotransferase | 2.50E-05 | -1.235 |
| *TFRC* | transferrin receptor | 4.00E-06 | -1.240 |
| *UQCC2* | ubiquinol-cytochrome c reductase complex assembly factor 2 | 4.00E-06 | -1.240 |
| *FAM216A* | family with sequence similarity 216 member A | 1.00E-05 | -1.240 |
| *MRPS7* | mitochondrial ribosomal protein S7 | 4.00E-06 | -1.245 |
| *TAGLN2* | transgelin 2 | 4.00E-06 | -1.245 |
| *URB2* | URB2 ribosome biogenesis 2 homolog (S. cerevisiae) | 6.00E-06 | -1.250 |
| *GIMAP7* | GTPase. IMAP family member 7 | 6.00E-06 | -1.250 |
| *OPLAH* | 5-oxoprolinase. ATP-hydrolysing | 6.00E-06 | -1.250 |
| *NT5C* | 5'. 3'-nucleotidase. cytosolic | 6.00E-06 | -1.250 |
| *PRPS2* | phosphoribosyl pyrophosphate synthetase 2 | 6.00E-06 | -1.250 |
| *HES1* | hes family bHLH transcription factor 1 | 2.00E-05 | -1.250 |
| *NREP* | neuronal regeneration related protein | 2.00E-05 | -1.250 |
| *E2F5* | E2F transcription factor 5 | 4.00E-06 | -1.255 |
| *NOP58* | NOP58 ribonucleoprotein | 6.00E-06 | -1.260 |
| *MRPL24* | mitochondrial ribosomal protein L24 | 4.00E-06 | -1.260 |
| *SH2D5* | SH2 domain containing 5 | 5.00E-06 | -1.260 |
| *CHRNA9* | cholinergic receptor nicotinic alpha 9 subunit | 8.00E-06 | -1.260 |
| *TYMS* | thymidylate synthetase | 5.00E-06 | -1.265 |
| *BOLA2* | bolA family member 2 | 7.00E-06 | -1.265 |
| *ID1* | inhibitor of DNA binding 1. HLH protein | 3.30E-05 | -1.265 |
| *TIMM23* | translocase of inner mitochondrial membrane 23 | 2.40E-05 | -1.265 |
| *PA2G4* | proliferation-associated 2G4 | 7.00E-06 | -1.270 |
| *GEMIN4* | gem nuclear organelle associated protein 4 | 4.00E-06 | -1.270 |
| *ETF1* | eukaryotic translation termination factor 1 | 4.00E-06 | -1.275 |
| *HHEX* | hematopoietically expressed homeobox | 5.00E-06 | -1.275 |
| *CIRH1A* | UTP4. small subunit processome component | 4.00E-06 | -1.280 |
| *SETSIP* | SET-like protein | 5.00E-06 | -1.285 |
| *TOMM5* | translocase of outer mitochondrial membrane 5 | 4.00E-06 | -1.285 |
| *SDCCAG3* | serologically defined colon cancer antigen 3 | 1.30E-05 | -1.290 |
| *RRM2* | ribonucleotide reductase regulatory subunit M2 | 4.00E-06 | -1.290 |
| *ELOVL6* | ELOVL fatty acid elongase 6 | 5.00E-06 | -1.295 |
| *MIF* | macrophage migration inhibitory factor | 4.00E-06 | -1.295 |
| *SLC37A4* | solute carrier family 37 member 4 | 4.00E-06 | -1.300 |
| *MRPS34* | mitochondrial ribosomal protein S34 | 6.00E-06 | -1.300 |
| *ABCB1* | ATP binding cassette subfamily B member 1 | 4.00E-06 | -1.305 |
| *PGAM4* | phosphoglycerate mutase family member 4 | 5.00E-06 | -1.305 |
| *ACAD9* | acyl-CoA dehydrogenase family member 9 | 5.70E-05 | -1.305 |
| *WDR77* | WD repeat domain 77 | 4.00E-06 | -1.310 |
| *AK2* | adenylate kinase 2 | 4.00E-06 | -1.310 |
| *CYBA* | cytochrome b-245 alpha chain | 8.00E-06 | -1.310 |
| *RUNX1* | runt related transcription factor 1 | 8.00E-06 | -1.310 |
| *LKAAEAR1* | LKAAEAR motif containing 1 | 5.00E-06 | -1.315 |
| *POLA2* | DNA polymerase alpha 2. accessory subunit | 7.00E-06 | -1.315 |
| *RRP15* | ribosomal RNA processing 15 homolog | 4.00E-06 | -1.315 |
| *NOP2* | NOP2 nucleolar protein | 4.00E-06 | -1.320 |
| *BCCIP* | BRCA2 and CDKN1A interacting protein | 6.00E-06 | -1.320 |
| *RPS7* | ribosomal protein S7 | 3.00E-06 | -1.325 |
| *PRR3* | proline rich 3 | 6.00E-06 | -1.325 |
| *POLR3G* | RNA polymerase III subunit G | 9.00E-06 | -1.325 |
| *SLC25A10* | solute carrier family 25 member 10 | 9.20E-05 | -1.325 |
| *HNRNPK* | heterogeneous nuclear ribonucleoprotein K | 6.00E-06 | -1.330 |
| *SFXN4* | sideroflexin 4 | 1.40E-05 | -1.330 |
| *TSR1* | TSR1. ribosome maturation factor | 2.20E-05 | -1.330 |
| *DDX39B* | DExD-box helicase 39B | 4.00E-06 | -1.330 |
| *APOBEC3B* | apolipoprotein B mRNA editing enzyme catalytic subunit 3B | 6.00E-06 | -1.330 |
| *FAR2* | fatty acyl-CoA reductase 2 | 4.00E-06 | -1.335 |
| *TMC5* | transmembrane channel like 5 | 8.50E-05 | -1.335 |
| *MRPL36* | mitochondrial ribosomal protein L36 | 4.00E-06 | -1.335 |
| *RPP40* | ribonuclease P/MRP subunit p40 | 4.00E-06 | -1.335 |
| *CA8* | carbonic anhydrase 8 | 1.40E-05 | -1.340 |
| *RGS19* | regulator of G protein signaling 19 | 4.00E-06 | -1.340 |
| *PRKCH* | protein kinase C eta | 4.00E-06 | -1.340 |
| *GAMT* | guanidinoacetate N-methyltransferase | 4.00E-06 | -1.340 |
| *SCD* | stearoyl-CoA desaturase | 4.00E-06 | -1.345 |
| *ALDH18A1* | aldehyde dehydrogenase 18 family member A1 | 4.00E-06 | -1.345 |
| *MYO1G* | myosin IG | 4.00E-06 | -1.345 |
| *CENPV* | centromere protein V | 5.00E-06 | -1.345 |
| *CCT5* | chaperonin containing TCP1 subunit 5 | 5.00E-06 | -1.345 |
| *POLR3K* | RNA polymerase III subunit K | 8.00E-06 | -1.345 |
| *SLC19A1* | solute carrier family 19 member 1 | 1.90E-05 | -1.350 |
| *EXOSC2* | exosome component 2 | 4.00E-06 | -1.355 |
| *NUP88* | nucleoporin 88 | 4.00E-06 | -1.355 |
| *PCYT2* | phosphate cytidylyltransferase 2. ethanolamine | 5.00E-06 | -1.360 |
| *SARS2* | seryl-tRNA synthetase 2. mitochondrial | 4.00E-06 | -1.365 |
| *TOMM40* | translocase of outer mitochondrial membrane 40 | 3.00E-06 | -1.365 |
| *IRAK1* | interleukin 1 receptor associated kinase 1 | 4.00E-06 | -1.365 |
| *FES* | FES proto-oncogene. tyrosine kinase | 4.00E-06 | -1.365 |
| *GCDH* | glutaryl-CoA dehydrogenase | 1.10E-05 | -1.365 |
| *TPI1* | triosephosphate isomerase 1 | 1.30E-05 | -1.365 |
| *BCKDK* | branched chain ketoacid dehydrogenase kinase | 5.00E-06 | -1.370 |
| *MTHFD1* | methylenetetrahydrofolate dehydrogenase. cyclohydrolase and formyltetrahydrofolate synthetase 1 | 7.00E-06 | -1.370 |
| *TXNDC5* | thioredoxin domain containing 5 | 7.00E-06 | -1.370 |
| *BCAT1* | branched chain amino acid transaminase 1 | 1.10E-05 | -1.370 |
| *GTF3A* | general transcription factor IIIA | 3.00E-06 | -1.380 |
| *S100A4* | S100 calcium binding protein A4 | 3.00E-06 | -1.380 |
| *DNPH1* | 2'-deoxynucleoside 5'-phosphate N-hydrolase 1 | 6.00E-06 | -1.380 |
| *LMO2* | LIM domain only 2 | 8.00E-06 | -1.380 |
| *RUVBL1* | RuvB like AAA ATPase 1 | 8.00E-06 | -1.380 |
| *LIMS2* | LIM zinc finger domain containing 2 | 3.90E-05 | -1.380 |
| *PPP1CB* | protein phosphatase 1 catalytic subunit beta | 4.00E-06 | -1.385 |
| *TBRG4* | transforming growth factor beta regulator 4 | 3.00E-06 | -1.385 |
| *VMA21* | VMA21. vacuolar ATPase assembly factor | 3.00E-06 | -1.385 |
| *TTLL12* | tubulin tyrosine ligase like 12 | 4.00E-06 | -1.385 |
| *CD320* | CD320 molecule | 5.00E-06 | -1.385 |
| *TNFRSF21* | TNF receptor superfamily member 21 | 2.90E-05 | -1.385 |
| *TSPAN4* | tetraspanin 4 | 3.00E-06 | -1.390 |
| *AMER1* | APC membrane recruitment protein 1 | 3.00E-06 | -1.390 |
| *FKBP4* | FK506 binding protein 4 | 6.00E-06 | -1.390 |
| *CPXM1* | carboxypeptidase X. M14 family member 1 | 9.00E-06 | -1.390 |
| *YDJC* | YdjC chitooligosaccharide deacetylase homolog | 1.40E-05 | -1.390 |
| *MARS2* | methionyl-tRNA synthetase 2. mitochondrial | 1.83E-04 | -1.390 |
| *C1QBP* | complement C1q binding protein | 3.00E-06 | -1.395 |
| *CDCA7* | cell division cycle associated 7 | 4.00E-06 | -1.395 |
| *EXOSC4* | exosome component 4 | 4.00E-06 | -1.395 |
| *RRP12* | ribosomal RNA processing 12 homolog | 7.00E-06 | -1.395 |
| *EBNA1BP2* | EBNA1 binding protein 2 | 3.00E-06 | -1.400 |
| *PRDX4* | peroxiredoxin 4 | 3.00E-06 | -1.400 |
| *NOP56* | NOP56 ribonucleoprotein | 4.00E-06 | -1.410 |
| *PNP* | purine nucleoside phosphorylase | 4.00E-06 | -1.410 |
| *ETS2* | ETS proto-oncogene 2. transcription factor | 5.00E-06 | -1.410 |
| *TFB2M* | transcription factor B2. mitochondrial | 6.00E-06 | -1.410 |
| *ZNF593* | zinc finger protein 593 | 2.10E-05 | -1.410 |
| *NR2C2AP* | nuclear receptor 2C2 associated protein | 4.00E-06 | -1.415 |
| *PGAM1* | phosphoglycerate mutase 1 | 8.00E-06 | -1.415 |
| *ATP9A* | ATPase phospholipid transporting 9A (putative) | 5.00E-06 | -1.420 |
| *TSPO* | translocator protein | 6.00E-06 | -1.420 |
| *CCR4* | C-C motif chemokine receptor 4 | 7.00E-06 | -1.420 |
| *TIGD5* | tigger transposable element derived 5 | 8.00E-06 | -1.420 |
| *ISOC2* | isochorismatase domain containing 2 | 3.00E-06 | -1.425 |
| *LTV1* | LTV1 ribosome biogenesis factor | 4.00E-06 | -1.425 |
| *P2RY11* | purinergic receptor P2Y11 | 6.00E-06 | -1.425 |
| *AIF1L* | allograft inflammatory factor 1 like | 3.00E-06 | -1.430 |
| *SEH1L* | SEH1 like nucleoporin | 3.00E-06 | -1.430 |
| *ATIC* | 5-aminoimidazole-4-carboxamide ribonucleotide formyltransferase/IMP cyclohydrolase | 3.00E-06 | -1.430 |
| *SNRPD1* | small nuclear ribonucleoprotein D1 polypeptide | 3.00E-06 | -1.435 |
| *TNFSF13B* | TNF superfamily member 13b | 8.00E-06 | -1.435 |
| *WDR46* | WD repeat domain 46 | 4.00E-06 | -1.435 |
| *WDR43* | WD repeat domain 43 | 3.00E-06 | -1.440 |
| *C16orf13* | methyltransferase like 26 | 4.00E-06 | -1.440 |
| *TRA2B* | transformer 2 beta homolog | 4.00E-06 | -1.440 |
| *ETV5* | ETS variant 5 | 4.00E-06 | -1.445 |
| *LIG4* | DNA ligase 4 | 1.70E-05 | -1.450 |
| *NME4* | NME/NM23 nucleoside diphosphate kinase 4 | 3.00E-06 | -1.450 |
| *UTP18* | UTP18. small subunit processome component | 1.50E-05 | -1.455 |
| *NDUFAF4* | NADH:ubiquinone oxidoreductase complex assembly factor 4 | 1.00E-05 | -1.465 |
| *PAGR1* | PAXIP1 associated glutamate rich protein 1 | 6.00E-06 | -1.470 |
| *SLC1A3* | solute carrier family 1 member 3 | 1.30E-05 | -1.470 |
| *RPL29* | ribosomal protein L29 | 3.00E-06 | -1.475 |
| *SOX4* | SRY-box 4 | 4.00E-06 | -1.475 |
| *LDHA* | lactate dehydrogenase A | 6.00E-06 | -1.475 |
| *EIF5A* | eukaryotic translation initiation factor 5A | 4.00E-06 | -1.480 |
| *GRWD1* | glutamate rich WD repeat containing 1 | 1.60E-05 | -1.480 |
| *TMEM177* | transmembrane protein 177 | 3.80E-05 | -1.480 |
| *IFRD2* | interferon related developmental regulator 2 | 3.00E-06 | -1.485 |
| *NKD2* | naked cuticle homolog 2 | 3.00E-06 | -1.485 |
| *PSME3* | proteasome activator subunit 3 | 3.00E-06 | -1.490 |
| *SLC39A3* | solute carrier family 39 member 3 | 3.00E-06 | -1.490 |
| *NRROS* | negative regulator of reactive oxygen species | 3.00E-06 | -1.490 |
| *NRXN2* | neurexin 2 | 4.00E-06 | -1.490 |
| *DNAAF5* | dynein axonemal assembly factor 5 | 4.00E-06 | -1.490 |
| *BZW2* | basic leucine zipper and W2 domains 2 | 3.00E-06 | -1.500 |
| *WDR74* | WD repeat domain 74 | 4.00E-06 | -1.500 |
| *FARSA* | phenylalanyl-tRNA synthetase alpha subunit | 3.00E-06 | -1.510 |
| *VEGFB* | vascular endothelial growth factor B | 3.00E-06 | -1.515 |
| *EEF2KMT* | eukaryotic elongation factor 2 lysine methyltransferase | 2.00E-06 | -1.520 |
| *GZMA* | granzyme A | 3.00E-06 | -1.520 |
| *SLC27A2* | solute carrier family 27 member 2 | 1.10E-05 | -1.520 |
| *MTHFD1L* | methylenetetrahydrofolate dehydrogenase (NADP+ dependent) 1 like | 6.00E-06 | -1.525 |
| *HNRNPAB* | heterogeneous nuclear ribonucleoprotein A/B | 4.00E-06 | -1.535 |
| *PPM1H* | protein phosphatase. Mg2+/Mn2+ dependent 1H | 3.00E-06 | -1.535 |
| *ZBTB21* | zinc finger and BTB domain containing 21 | 4.00E-06 | -1.535 |
| *POLR1C* | RNA polymerase I subunit C | 2.00E-06 | -1.540 |
| *CTSC* | cathepsin C | 1.10E-05 | -1.540 |
| *HK2* | hexokinase 2 | 4.00E-06 | -1.545 |
| *OPRL1* | opioid related nociceptin receptor 1 | 3.00E-06 | -1.550 |
| *UBA1* | ubiquitin like modifier activating enzyme 1 | 6.00E-06 | -1.550 |
| *WDR12* | WD repeat domain 12 | 2.00E-06 | -1.555 |
| *ACKR3* | atypical chemokine receptor 3 | 3.00E-06 | -1.555 |
| *NKG7* | natural killer cell granule protein 7 | 3.00E-06 | -1.555 |
| *STC1* | stanniocalcin 1 | 4.00E-06 | -1.555 |
| *MMACHC* | methylmalonic aciduria (cobalamin deficiency) cblC type. with homocystinuria | 4.00E-06 | -1.555 |
| *CHI3L2* | chitinase 3 like 2 | 5.00E-06 | -1.560 |
| *HNRNPA1* | heterogeneous nuclear ribonucleoprotein A1 | 6.50E-05 | -1.560 |
| *BCL11B* | B cell CLL/lymphoma 11B | 3.00E-06 | -1.565 |
| *WDR18* | WD repeat domain 18 | 2.00E-06 | -1.575 |
| *CHCHD4* | coiled-coil-helix-coiled-coil-helix domain containing 4 | 3.00E-06 | -1.580 |
| *DKC1* | dyskerin pseudouridine synthase 1 | 3.00E-06 | -1.580 |
| *PTPRC* | protein tyrosine phosphatase. receptor type C | 5.00E-06 | -1.580 |
| *NAA15* | N(alpha)-acetyltransferase 15. NatA auxiliary subunit | 3.00E-06 | -1.590 |
| *CDK4* | cyclin dependent kinase 4 | 3.00E-06 | -1.590 |
| *RASGRP2* | RAS guanyl releasing protein 2 | 9.00E-06 | -1.590 |
| *DUT* | deoxyuridine triphosphatase | 2.00E-06 | -1.600 |
| *RPL6* | ribosomal protein L6 | 3.00E-06 | -1.605 |
| *IL7R* | interleukin 7 receptor | 3.00E-06 | -1.605 |
| *PAK1IP1* | PAK1 interacting protein 1 | 2.00E-06 | -1.610 |
| *CD59* | CD59 molecule (CD59 blood group) | 2.00E-06 | -1.615 |
| *RPS24* | ribosomal protein S24 | 3.00E-06 | -1.620 |
| *LYL1* | LYL1. basic helix-loop-helix family member | 2.00E-06 | -1.625 |
| *CCND2* | cyclin D2 | 3.00E-06 | -1.625 |
| *PTMA* | prothymosin. alpha | 5.00E-06 | -1.625 |
| *KISS1R* | KISS1 receptor | 4.00E-06 | -1.630 |
| *BRIX1* | BRX1. biogenesis of ribosomes | 3.00E-06 | -1.635 |
| *FAAP100* | Fanconi anemia core complex associated protein 100 | 3.00E-06 | -1.635 |
| *BIK* | BCL2 interacting killer | 3.00E-06 | -1.635 |
| *MZB1* | marginal zone B and B1 cell specific protein | 3.00E-06 | -1.635 |
| *MTFP1* | mitochondrial fission process 1 | 3.00E-06 | -1.640 |
| *CLEC11A* | C-type lectin domain containing 11A | 6.00E-06 | -1.640 |
| *SLC29A1* | solute carrier family 29 member 1 (Augustine blood group) | 2.00E-06 | -1.645 |
| *CELSR3* | cadherin EGF LAG seven-pass G-type receptor 3 | 2.00E-06 | -1.665 |
| *DHX37* | DEAH-box helicase 37 | 2.00E-06 | -1.665 |
| *DHX33* | DEAH-box helicase 33 | 3.00E-06 | -1.665 |
| *PDSS1* | decaprenyl diphosphate synthase subunit 1 | 3.00E-06 | -1.665 |
| *C10orf2* | twinkle mtDNA helicase | 2.00E-06 | -1.680 |
| *ATP5G1* | ATP synthase membrane subunit c locus 1 | 2.00E-06 | -1.680 |
| *UCK2* | uridine-cytidine kinase 2 | 2.00E-06 | -1.685 |
| *DBNL* | drebrin like | 3.00E-06 | -1.685 |
| *MCM7* | minichromosome maintenance complex component 7 | 3.00E-06 | -1.690 |
| *SUMO3* | small ubiquitin-like modifier 3 | 3.00E-06 | -1.690 |
| *MRTO4* | MRT4 homolog. ribosome maturation factor | 2.00E-06 | -1.700 |
| *DCTPP1* | dCTP pyrophosphatase 1 | 2.00E-06 | -1.700 |
| *MATK* | megakaryocyte-associated tyrosine kinase | 2.00E-06 | -1.710 |
| *AEN* | apoptosis enhancing nuclease | 2.00E-06 | -1.720 |
| *FKBP11* | FK506 binding protein 11 | 2.00E-06 | -1.720 |
| *SRM* | spermidine synthase | 3.00E-06 | -1.720 |
| *SPRY1* | sprouty RTK signaling antagonist 1 | 3.00E-06 | -1.725 |
| *EMG1* | EMG1. N1-specific pseudouridine methyltransferase | 2.00E-06 | -1.730 |
| *ISPD* | isoprenoid synthase domain containing | 3.00E-06 | -1.735 |
| *TNFRSF1B* | TNF receptor superfamily member 1B | 3.00E-06 | -1.735 |
| *RPL8* | ribosomal protein L8 | 3.00E-06 | -1.745 |
| *IFITM2* | interferon induced transmembrane protein 2 | 3.00E-06 | -1.745 |
| *ALDH1A2* | aldehyde dehydrogenase 1 family member A2 | 2.00E-06 | -1.750 |
| *PRMT1* | protein arginine methyltransferase 1 | 2.00E-06 | -1.760 |
| *POLR3H* | RNA polymerase III subunit H | 4.00E-06 | -1.765 |
| *DDX21* | DExD-box helicase 21 | 2.00E-05 | -1.765 |
| *CHORDC1* | cysteine and histidine rich domain containing 1 | 3.00E-06 | -1.775 |
| *PAICS* | phosphoribosylaminoimidazole carboxylase and phosphoribosylaminoimidazolesuccinocarboxamide synthase | 2.00E-06 | -1.780 |
| *GTPBP4* | GTP binding protein 4 | 2.00E-06 | -1.785 |
| *BCL11A* | B cell CLL/lymphoma 11A | 3.00E-06 | -1.800 |
| *EXOSC5* | exosome component 5 | 2.00E-06 | -1.805 |
| *RRP7A* | ribosomal RNA processing 7 homolog A | 2.00E-06 | -1.805 |
| *RRS1* | ribosome biogenesis regulator homolog | 2.40E-05 | -1.810 |
| *HHIP* | hedgehog interacting protein | 2.00E-06 | -1.815 |
| *ABCE1* | ATP binding cassette subfamily E member 1 | 2.00E-06 | -1.820 |
| *ILF3* | interleukin enhancer binding factor 3 | 1.70E-05 | -1.825 |
| *HMBS* | hydroxymethylbilane synthase | 2.00E-06 | -1.840 |
| *ABCB10* | ATP binding cassette subfamily B member 10 | 2.00E-06 | -1.845 |
| *GART* | phosphoribosylglycinamide formyltransferase. phosphoribosylglycinamide synthetase. phosphoribosylaminoimidazole synthetase | 3.00E-06 | -1.845 |
| *BYSL* | bystin like | 3.00E-06 | -1.845 |
| *KIAA0020* | pumilio RNA binding family member 3 | 2.00E-06 | -1.850 |
| *IRX3* | iroquois homeobox 3 | 2.00E-06 | -1.850 |
| *NME1* | NME/NM23 nucleoside diphosphate kinase 1 | 2.00E-06 | -1.850 |
| *TSPAN7* | tetraspanin 7 | 2.00E-06 | -1.860 |
| *NOLC1* | nucleolar and coiled-body phosphoprotein 1 | 3.00E-06 | -1.875 |
| *MYB* | MYB proto-oncogene. transcription factor | 2.00E-06 | -1.880 |
| *FAM195A* | MAPK regulated corepressor interacting protein 2 | 5.00E-06 | -1.880 |
| *RPS15* | ribosomal protein S15 | 2.00E-06 | -1.920 |
| *METTL1* | methyltransferase like 1 | 2.00E-06 | -1.980 |
| *SLC2A6* | solute carrier family 2 member 6 | 2.00E-06 | -2.005 |
| *MRPL20* | mitochondrial ribosomal protein L20 | 2.00E-06 | -2.015 |
| *PLD6* | phospholipase D family member 6 | 2.00E-06 | -2.020 |
| *PTGES2* | prostaglandin E synthase 2 | 2.00E-06 | -2.030 |
| *SELL* | selectin L | 4.00E-06 | -2.030 |
| *PHB* | prohibitin | 2.00E-06 | -2.035 |
| *SRSF7* | serine and arginine rich splicing factor 7 | 2.00E-06 | -2.040 |
| *PPRC1* | peroxisome proliferator-activated receptor gamma. coactivator-related 1 | 6.00E-06 | -2.070 |
| *NOP16* | NOP16 nucleolar protein | 2.00E-06 | -2.080 |
| *PUS7* | pseudouridylate synthase 7 (putative) | 2.00E-06 | -2.105 |
| *CCR7* | C-C motif chemokine receptor 7 | 2.00E-06 | -2.115 |
| *GPATCH4* | G-patch domain containing 4 | 2.00E-06 | -2.135 |
| *MRPL12* | mitochondrial ribosomal protein L12 | 3.00E-06 | -2.145 |
| *SNHG15* | small nucleolar RNA host gene 15 | 2.00E-06 | -2.170 |
| *SCG2* | secretogranin II | 2.00E-06 | -2.180 |
| *PUS1* | pseudouridylate synthase 1 | 2.00E-06 | -2.185 |
| *GNL3* | G protein nucleolar 3 | 2.00E-06 | -2.190 |
| *LYAR* | Ly1 antibody reactive | 2.00E-06 | -2.195 |
| *TCF4* | transcription factor 4 | 2.00E-06 | -2.205 |
| *FAM69B* | family with sequence similarity 69 member B | 2.00E-06 | -2.210 |
| *RANBP1* | RAN binding protein 1 | 2.00E-06 | -2.225 |
| *LDLR* | low density lipoprotein receptor | 2.00E-06 | -2.225 |
| *MFSD3* | major facilitator superfamily domain containing 3 | 2.00E-06 | -2.255 |
| *UHRF1* | ubiquitin like with PHD and ring finger domains 1 | 2.00E-06 | -2.255 |
| *SOSTDC1* | sclerostin domain containing 1 | 2.00E-06 | -2.305 |
| *UNG* | uracil DNA glycosylase | 2.00E-06 | -2.310 |
| *DNAAF3* | dynein axonemal assembly factor 3 | 2.00E-06 | -2.325 |
| *CCDC86* | coiled-coil domain containing 86 | 2.00E-06 | -2.355 |
| *FASN* | fatty acid synthase | 2.00E-06 | -2.355 |
| *NKX2-5* | NK2 homeobox 5 | 2.00E-06 | -2.360 |
| *PFAS* | phosphoribosylformylglycinamidine synthase | 2.00E-06 | -2.375 |
| *VARS* | valyl-tRNA synthetase | 2.00E-06 | -2.555 |
| *MYCN* | MYCN proto-oncogene. bHLH transcription factor | 3.00E-06 | -2.590 |
| *LRRC26* | leucine rich repeat containing 26 | 2.00E-06 | -2.765 |
| *SYNCRIP* | synaptotagmin binding cytoplasmic RNA interacting protein | 1.00E-06 | -2.805 |
| *PVRIG* | PVR related immunoglobulin domain containing | 1.00E-06 | -2.820 |
| *IGLL3P* | immunoglobulin lambda like polypeptide 3. pseudogene | 2.00E-06 | -2.995 |
| *LINC00977* | long intergenic non-protein coding RNA 977 | 2.00E-06 | -3.005 |
| *IGLL1* | immunoglobulin lambda like polypeptide 5 | 2.00E-06 | -3.110 |
| *MYC* | MYC proto-oncogene. bHLH transcription factor | 1.00E-06 | -4.260 |

Chipster analysis identified 731 significantly deregulated genes in CEM/ADR5000 treated cells.
